# The *Chlamydia psittaci* Genome: A Comparative Analysis of Intracellular Pathogens

**DOI:** 10.1371/journal.pone.0035097

**Published:** 2012-04-10

**Authors:** Anja Voigt, Gerhard Schöfl, Hans Peter Saluz

**Affiliations:** 1 Leibniz-Institute for Natural Product Research and Infection Biology, Jena, Germany; 2 Friedrich Schiller University, Jena, Germany; University of California Merced, United States of America

## Abstract

**Background:**

*Chlamydiaceae* are a family of obligate intracellular pathogens causing a wide range of diseases in animals and humans, and facing unique evolutionary constraints not encountered by free-living prokaryotes. To investigate genomic aspects of infection, virulence and host preference we have sequenced *Chlamydia psittaci*, the pathogenic agent of ornithosis.

**Results:**

A comparison of the genome of the avian *Chlamydia psittaci* isolate 6BC with the genomes of other chlamydial species, *C. trachomatis*, *C. muridarum*, *C. pneumoniae*, *C. abortus*, *C. felis* and *C. caviae*, revealed a high level of sequence conservation and synteny across taxa, with the major exception of the human pathogen *C. trachomatis*. Important differences manifest in the polymorphic membrane protein family specific for the *Chlamydiae* and in the highly variable chlamydial plasticity zone. We identified a number of *psittaci*-specific polymorphic membrane proteins of the G family that may be related to differences in host-range and/or virulence as compared to closely related *Chlamydiaceae*. We calculated non-synonymous to synonymous substitution rate ratios for pairs of orthologous genes to identify putative targets of adaptive evolution and predicted type III secreted effector proteins.

**Conclusions:**

This study is the first detailed analysis of the *Chlamydia psittaci* genome sequence. It provides insights in the genome architecture of *C. psittaci* and proposes a number of novel candidate genes mostly of yet unknown function that may be important for pathogen-host interactions.

## Introduction

*Chlamydia psittaci* is the pathogenic agent of ornithosis or psittacosis, a primarily avian respiratory disease with sizeable impact on poultry farming and bird breeding economic returns [1]. It belongs to the family *Chlamydiaceae*, a small group of extremely successful obligate intracellular pathogens that efficiently colonize mucosal surfaces and thrive within a wide variety of animal hosts, including humans [1].

Although birds are the primary targets of *C. psittaci* infections [2], transmissions from birds to humans have been reported, especially where humans come into close contact with infected birds on a regular basis, as in the case of veterinarians, poultry farmers, or bird breeders [3]–[5]. Moreover, *C. psittaci* has been isolated from a variety of other mammalian hosts, including cattle and other ruminants, horses, and pigs [1], [6].

Other medically important members of the family *Chlamydiaceae* are the human-specific *C. trachomatis* and the wide-host-range *C. pneumoniae*. Worldwide, *C. trachomatis* is a leading cause of sexually transmitted bacterial diseases and ocular infections (trachoma), potentially leading to blindness [7]. *C. pneumoniae* is transmitted by respiratory droplets and the causative agent of an atypical pneumonia and other acute respiratory illnesses [8].

Currently, within *Chlamydiaceae* a total of nine species are organized in the single genus *Chlamydia*: *C. trachomatis*, *C. muridarum*, *C. pneumoniae*, *C. pecorum*, *C. suis*, *C. abortus*, *C. felis*, *C. caviae*, and *C. psittaci*. All *Chlamydiaceae* need to infect eukaryote host cells for replication. Despite major differences in host range, tissue tropism, and disease pathology they all share a characteristic biphasic developmental cycle unique among prokaryotes [9], [10]. The infectious chlamydial form, the elementary body (EB), enters the eukaryotic cell and becomes internalized in a vacuole in the cytoplasm of the host cell. In this so called inclusion, the EB differentiates into a non-infectious, metabolically active form, the reticulate body (RB). The RB multiplies by binary fission [1], [10]. Depending on the strain, two to three days after infection the RBs transform back into EBs, which get released by lysis of the host cell or exocytosis [9].

As a consequence of this life style, the arguably most important driving force for the evolution of these pathogens is the interaction with their peculiar ecological niche, the inclusion within the cytoplasm of a eukaryotic host. Specific genomic manifestations of the obligate intracellular lifestyle have long been recognized, e.g. a strong trend towards reduced genome sizes, a dramatical reduction of effective population size relative to environmental bacteria making purifying selection comparatively less efficient, extreme sequence divergence in proteins that mediate the interaction with the host environment such as outer surface proteins and secretion systems (reviewed in [11]). Hence, a comparison of genes involved either directly or indirectly in interactions with the host cell is most likely to shed light on the evolution of the intracellular life style of the *Chlamydiaceae*, and their adaptation to different eukaryote hosts.

Currently, relatively little is known regarding the chlamydial factors involved in virulence, host interaction, or host specificity. Genes for which functions in relation to niche adaptation have been implicated are mainly (i) the polymorphic membrane proteins (pmps), a large family of proteins probably unique to the phylum *Chlamydiae* [12], [13], and considered to be important in adhesion of the EB to the host cell, molecular transport, and cell wall associated functions [14]; (ii) genes located in the chlamydial “plasticity zone”, a distinct region close to the terminus of replication, characterized by unusually high levels of inter- and intraspecific polymorphism [15]; and (iii) the so called type III effector proteins, a highly variable class of proteins potentially secreted into the host cell by the molecular machinery of the type III secretion (T3S) apparatus.

The T3S apparatus and the effectors translocated by it form a sophisticated mechanism of bacterial pathogenesis found in a number of Gram-negative bacteria. It is characterized by the direct translocation of effector proteins into the host cytoplasm to mediate colonization and parasitation of susceptible hosts [16], [17]. T3S effector proteins display little sequence homology across species. Although the N-terminal regions of T3S effectors show unusual amino acid compositions (e.g. [18]) no unambiguous common motif among different T3S signal sequences has been established, making the computational prediction of putative T3S effectors a difficult challenge [18], [19].

Whole-genome comparison between phylogenetically distant chlamydial species that parasitize a range of host species, vary in their host specificity and pathogenicity can provide a foundation from which to comprehend factors involved in chlamydial niche adaptation. For this study we sequenced the genome of the pathogenic avian type strain *Chlamydia psittaci* 6BC. Meanwhile two additional *C. psittaci* genome sequences have become available [20], [21]. This study represents the first detailed analysis of the *C. psittaci* genome sequence, however. Our results show a typical chlamydial genome with a coding capacity of 967 CDSs. The comparative analysis of the *C. psittaci* genome with those of other *Chlamydiaceae* confirms the exceptional roles of the family of polymorphic membrane proteins and the chlamydial plasticity zone as source of most interspecies variation. The prediction of putative type III secreted effectors and a genome-wide analyses of non-synonymous to synonymous substitution rate ratios yield a number of novel candidate genes likely involved in host-pathogen interactions and adaptive divergence between *C. psittaci* and their relatives.

## Results and Discussion

### Genome sequence of the avian isolate 6BC of *C. psittaci*

*Chlamydia psittaci* 6BC possesses a single circular chromosome of 1.172 Mb and a plasmid of 7553 bp. The bacterial chromosome is predicted to contain 967 coding sequences (CDSs) and the plasmid is predicted to harbour eight coding sequences. 26% of the CDSs are annotated as encoding hypothetical products. The general features of the *C. psittaci* genome in comparison to other sequenced chlamydial genomes are summarized in Table 1.

**Table 1:** Summary of *Chlamydiaceae* genome features.

	*C. psittaci* 6BC	*C. abortus* S26/3	*C. felis* Fe/C-56	*C. caviae* GPIC	*C. pneumoniae* LPCoLN	*C. trachomatis* L2/434/Bu
Genome size [bp]	1,171,660	1,144,377	1,166,239	1,173,390	1,241,020	1,038,842
No. of CDSs	967	932	1005	998	1097	874
Coding density [%]	89.39	87.62	91.22	89.42	89.44	89.09
Average gene size [bp]	1083	1075	1059	1051	1012	1059
% G+C content	39.06	39.87	39.38	39.22	40.55	41.33
% G+C of CDSs	39.46	40.24	39.95	39.82	41.26	41.66
tRNA	38	38	38	38	38	37
rRNA operons	1	1	1	1	1	2

**Figure 1:**
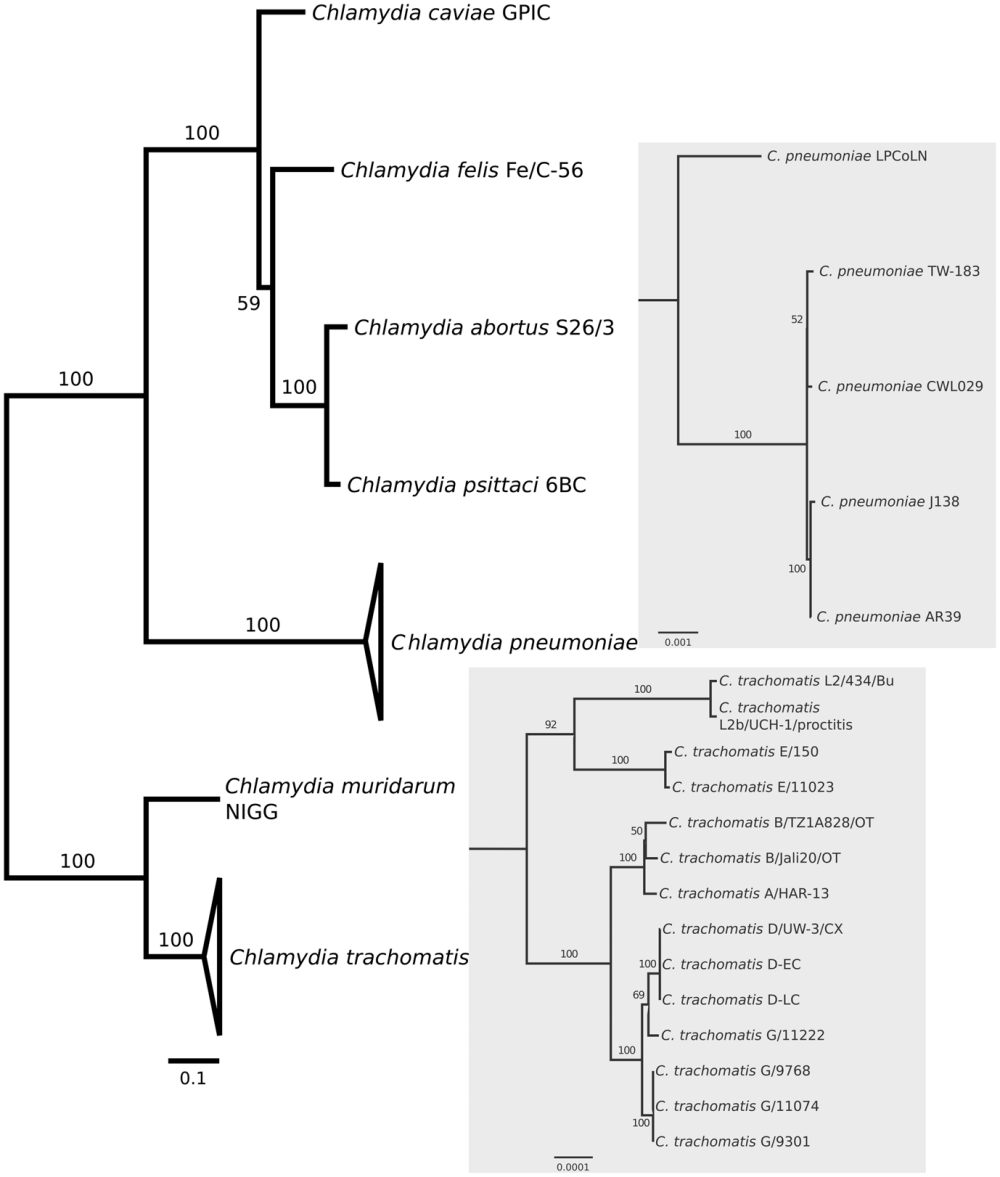
Phylogenomic relationships among sequenced chlamydial genomes. The maximum-likelihood tree is based on 100 randomly chosen conserved orthologous genes. Bootstrap values are displayed at the branches. The panels show the within-species phylogenetic relationships of the sequenced genomes of *Chlamydia pneumoniae* (upper panel) and *C. trachomatis* (lower panel).

A phylogenetic tree was reconstructed for all species within *Chlamydiaceae* for which full-length genomic sequences have become available, including intraspecific variants (Figure 1). The inferred topology is consistent with previous phylogenies of the *Chlamydiaceae* (e.g. [22], [23]), and shows the close relationship of *C. psittaci* with the three chlamydial species originally considered as the “mammalian” *Chlamydia psittaci* abortion, feline, and Guinea pig strains (i.e., *C. abortus*, *C. felis*, and *C. caviae*; here referred to as “*psittaci*-group”).

**Figure 2:**
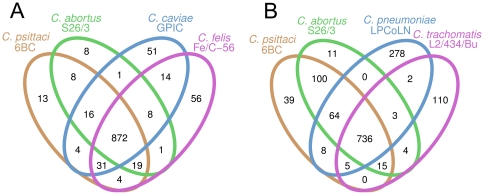
Venn diagrams showing the numbers of predicted CDSs that are unique or shared among two or more taxa. **A.** Number of shared genes among the closely related *C. psittaci*, *C. abortus*, *C. caviae*, and *C. felis*; **B.** number of shared genes among the wider range of *Chlamydiaceae*. Pseudogenes were scored as absent in this analysis.

**Figure 3:**
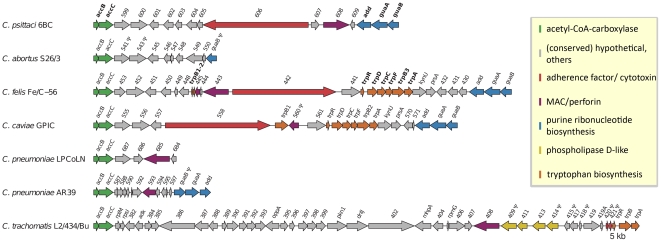
Comparison of the plasticity zone of *C. psittaci* 6BC, *C. abortus* S26/3, *C. felis* Fe/C-56, *C. caviae* GPIC, *C. pneumoniae* LPCoLN and AR39, and *C. trachomatis* L2/434/Bu. Genes are labelled with the published locus tag numbers. Colour-coded genes are discussed in the text. Pseudogenes are marked by 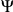.

A comparison of the genomes of the *psittaci*-group shows that all four species are highly conserved in terms of size, in the number of coding sequences and in nucleotide composition (Table 1). The degree of conservation is illustrated by a comparison of the coding capacity of these genomes: 872 of the predicted CDSs of *C. psittaci* are shared among all four species of the *psittaci*-group (with a total CDS count ranging from 967 to 1005), 82 are divergent, and only 13 genes are unique to *C. psittaci* (Figure 2A). Notably, six of the 13 *C. psittaci* genes identified as having no significant homology to any other chlamydial species encode polymorphic membrane proteins (pmps) of the G family, most others encode hypothetical proteins with unknown functions (Table 2). Four of these unique genes (CPSIT_0306, CPSIT_0429, CPSIT_0605, and CPSIT_0846) are predicted type III secreted effector proteins (Table 3 and Table S1). Three of these genes are located in the hypervariable chlamydial plasticity zone (CPSIT_603, CPSIT_605, and CPSIT_607, Figure 3), a region at the terminus of replication that contains an array of chlamydial niche-specific genes putatively important in host-specific interactions [24].

**Table 2:** Predicted CDSs unique to *C. psittaci* 6BC.

CDS	Product description
CPSIT_0309	polymorphic membrane protein, G family
CPSIT_0310	polymorphic membrane protein, G family
CPSIT_0311^1^	polymorphic membrane protein, G family
CPSIT_0312^1^	polymorphic membrane protein, G family
CPSIT_0316^1^	polymorphic membrane protein, G family
CPSIT_0330	putative outer membrane protein
CPSIT_0429^1^	hypothetical protein
CPSIT_0603	conserved hypothetical protein
CPSIT_0605	hypothetical protein
CPSIT_0607	hypothetical protein
CPSIT_0661	conserved domain protein
CPSIT_0668	polymorphic membrane protein, G family
CPSIT_0846^1^	putative TMH-family membrane protein

1 predicted T3S effector protein.

**Table 3:** Comparison of predicted type III secreted proteins of *C. psittaci* 6BC with orthologs in other *Chlamydiaceae*.

*C. psittaci* 6BC							
ORF	SVM value	Annotation	*C. abortus* S26/3	*C. felis* Fe/C-56	*C. caviae* GPIC	*C. pneumoniae* LPCoLN	*C. trachomatis* L2/434/Bu
CPSIT0357	1.957	hypothetical protein	-	678*	325*	-	-
CPSIT0192	1.655	orthologous to *C. trachomatis* TARP	167*	837*	170*	1089*	0716*
CPSIT0429	1.309	hypothetical protein	-	-	-	-	-
CPSIT0580	1.224	putative inner membrane protein	522*	472*	536*	-	-
CPSIT0074	1.221	conserved hypothetical serine rich protein	063*	942*	062*	-	-
CPSIT0422	1.152	conserved hypothetical protein	-	618*	390	-	-
CPSIT0397	1.146	conserved hypothetical protein	357*	640*	367*	0938*	0528
CPSIT0463	1.109	putative inner membrane protein	412*	582	426	-	-
CPSIT0421	1.087	conserved hypothetical protein	376*	619*	389*	-	-
CPSIT0532	1.087	inclusion membrane protein B	477*	516*	491	0799*	0484*
CPSIT0997	1.080	putative inner membrane protein	910*	073*	941*	0230*	0828*
CPSIT0606	1.061	adherence factor	550	442	558*	-	-
CPSIT0245	1.054	carbohydrate isomerase	215*	788*	219*	1041*	0656
CPSIT0757	1.050	dihydrodipicolinate reductase	680*	303*	715*	0474*	0618
CPSIT0594	1.005	inclusion membrane protein A	536*	458*	550*	-	-
CPSIT0220	0.971	cyclodiphosphate synthase	191*	812*	195*	01062	0693
CPSIT0431	0.952	putative membrane protein	-	610	397*	-	-
CPSIT0846	0.896	putative TMH-family/IncA-family protein	766	-	-	-	-
CPSIT0844	0.864	putative TMH-family/IncA-family protein	764*	218	797*	-	-
CPSIT0689	0.858	conserved hypothetical protein	618	-	647	-	-
CPSIT0933	0.846	putative membrane protein	852	129*	-	0291*	-
CPSIT0314	0.829	polymorphic membrane protein, G family	283*	719*	-	-	-
CPSIT0749	0.769	conserved hypothetical protein	673*	-	707	-	-
CPSIT0296	0.746	hypothetical serine rich protein	264*	738*	270*	-	-
CPSIT0785	0.737	conserved hypothetical serine rich protein	706*	277*	739*	0448*	0238*
CPSIT0853	0.707	putative membrane protein	773*	214*	803*	-	-
CPSIT0602	0.706	conserved hypothetical protein	546	448	-	-	-
CPSIT0962	0.704	flagellar biosynthesis/type III secretory pathway	876*	106*	908*	0265*	0087
CPSIT0490	0.677	conserved hypothetical serine rich protein	437*	556*	451*	0841*	0338*
CPSIT1042	0.648	deoxyribonucleotide triphosphate pyrophosphatase	952*	031*	982*	0180	0869
CPSIT0828	0.635	DNA recombination protein	747*	234*	779*	0407	0197
CPSIT0760	0.573	hypothetical membrane protein	683*	300*	718	-	-
CPSIT0656	0.552	putative integral membrane protein	588*	388*	616	0664*	402*
CPSIT0974	0.549	trigger factor	887*	095*	919*	0254*	0076
CPSIT0313	0.545	polymorphic membrane protein, G family	282*	721*	284*	0958	-
CPSIT1054	0.540	5-formyltetrahydrofolate cyclo-ligase	964*	019*	994*	0169	017
CPSIT0555	0.528	putative inner membrane protein	500*	494*	513	-	-
CPSIT0930	0.517	tRNA(Uracil-5-)-methyltransferase	849*	132*	883	0294*	0111
CPSIT0461	0.513	hypothetical protein	411	-	425*	-	-
CPSIT0767	0.506	3-phosphoshikimate 1-carboxyvinyltransferase	690*	293*	723*	0466*	0620*

Orthologs predicted to be type three secreted are represented with *.

Only genes with an SVM decision value >0.5 are reported as candidate type III secreted proteins [18].

ORFs hightlighted in gray have also been predicted as T3SEs by EffectiveT3 ( http://effectors.org ).

When the more divergent species *C. pneumoniae* and *C. trachomatis* were included in the comparison, 736 of the predicted CDSs (ranging from 874 to 1097) were common to all *Chlamydiaceae* (Figure 2B). 64 genes were shared exclusively by *C. psittaci*, *C. abortus*, and *C. pneumoniae*, all members of the recently deprecated genus *Chlamydophila* [23], [25] (Figure 2B).

**Figure 4:**
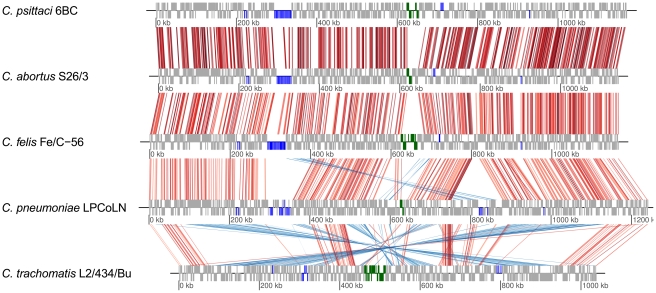
Global genome comparison between the *C. psittaci* 6BC, *C. abortus* S26/3, *C. felis* Fe/C-56, *C. pneumoniae* LPCoLN, and *C. trachomatis* L2/434/Bu genomes. The figure shows orthologous matches visualized using genoPlotR (compare Methods). The grey tick marks above and below the sequence lines represent the predicted CDSs on the plus strand and the minus strand of the genomes, respectively. Colour-marked are (blue) members of the polymorphic membrane protein family (pmp) and (green) the position of the plasticity zone (PZ). The red lines connecting genome lines represent direct orthologous matches. The blue lines represent reversed matches. Darker colours correspond to a higher bit scores.

Whole-genome comparisons with the other published *Chlamydiaceae* genomes show that the *C. psittaci* 6BC genome is essentially syntenic to sequences from *C. abortus*, *C. felis*, and *C. pneumoniae* (Figure 4).

A high degree of genomic rearrangement, however, becomes apparent with respect to *C. trachomatis* (Figure 4). Major deviations from synteny and/or sequence conservation are found in a hypervariable region near the replication terminus that has been termed “plasticity zone” (PZ) [15] and among the family of polymorphic membrane protein (pmp) genes [26] (Figure 4). These regions are suspected to harbour key genomic correlates to species-specific adaptation to different environmental niches, differential tissue tropism and differences in virulence and pathogenicity [27]. Additionally, putative virulence factors, e.g. proteins mediating the chlamydial attachment to the host cell, or those related to the chlamydial inclusion membrane and development, may play a crucial role in niche adaptation, such as members of Inc/Tmh protein family (inclusion membrane proteins and transmembrane head proteins) and type III secreted effector proteins (T3SE) [15], [28].

It is interesting to note, that a recent revision of the taxonomy of the *Chlamydiaceae* saw the genus *Chlamydia* retained as the sole genus in the family [25], thus deprecating the usage of the genus *Chlamydophila* introduced by Everett et al. in 1999 [29]. However, the phylogenetic analysis based on concatenated protein sequences (Figure 1), the numbers of shared orthologous genes (Figure 2B), and the level of synteny between genomes (Figure 4) all clearly do support the separation of *Chlamydiaceae* into two groups that correspond to the former genera, *Chlamydia* and *Chlamydophila*. Thus, irrespective of whether by any formal criteria *Chlamydophila* can be considered a genus, the label does remain useful as a moniker for an evolutionarily distinct branch within the *Chlamydiaceae*.

### Plasticity zone and polymorphic membrane proteins

The size and organisation of the plasticity zone differs substantially among *Chlamydiaceae* (Figure 3). This high level of genetic diversity is thought to correspond to rapid evolution of a set of putative virulence factors which have accumulated in chlamydial PZs. Thus, a number of the genes contained in the chlamydial PZs have been linked to host-pathogen interactions/pathogenesis, e.g., a MAC/perforin domain gene [30], a cytotoxin gene similar to the EHEC adherence factor and clostridial large cytotoxins [24], [31], tryptophan biosynthesis genes [31], [32], or phospholipase D family enzymes [33].

In *C. psittaci* 6BC the PZ spans about 29 kb and encodes 16 genes. It has less gene content than the respective plasticity zones of *C. caviae* and *C. felis* (22 and 29 genes) but is larger than the PZ of *C. abortus* (11 genes). These differences arise because *C. psittaci* and *C. abortus* lack the complete tryptophan biosynthesis operon (*trpABFCDR*, *kynU*, *prsA*) present in the *C. felis* and *C. caviae* genomes. In contrast to *C. abortus*, *C. psittaci* shares a putatively functional 10074 bp EHEC-like adherence factor (CPSIT_0606), a 2466 bp MAC/perforin domain gene (CPSIT_0608), and a *guaAB-add* cluster serving purine nucleotide interconversion with *C. caviae* and *C. felis* (Figure 3). Three of the hypothetical proteins in the *C. psittaci* PZ (CPSIT_0603, CPSIT_0605, CPSIT_0607) are predicted to be type III secreted effector proteins.

Among the recognizable putative toxin genes of the PZ, a full-length version of a chlamydial MAC/perforin domain gene was present in the *C. psittaci*, *C. felis*, *C. pneumoniae* koala LPCoLN and *C. trachomatis* isolates. A MAC/perforin domain protein was absent from the *C. abortus* genome [27], and showed frame disruptions in *C. caviae* and the *C. pneumoniae* human isolates [34] (Figure 3). The *C. psittaci* MAC/perforin also shows a MIR (protein nannosyltransferase, IP3R and RyR) domain indicative of a possible ligand transferase function. It shares this feature only with the more distant orthologs in *C. pneumoniae* koala LPCoLN and *C. trachomatis*, but not *C. felis* [34].

Many eukaryotic MAC/perforin proteins function as membrane perforation proteins [35], and are known to play important roles in plant and animal immune response to bacterial infections [36]. The functional role of the MAC/perforin domain genes in *Chlamydiae* is unclear. It has been suggested that proteins with MAC/perforin structures might help in evading the host immune response through structural mimicry, e.g. if the assembly of a host MAC/perforin complex is prevented by pathogen-derived MAC/perforin molecules present on the pathogen's cell surface [37], [38]. It is likely that the chlamydial MAC/perforin genes were obtained by horizontal gene transfer from an eukaryotic source [39], [40].

A full-length lymphostatin/EHEC adherence factor is present only in *C. psittaci*, *C. felis*, and *C. caviae* (Figure 3) as well as in *C. muridarum* (three orthologs [15]) and *C. pecorum* [41]. This cytotoxin is generally absent from the human-specific pathogens *C. trachomatis* (except for gene fragments [42], [43]) and *C. pneumoniae* [15], [34].

**Figure 5:**
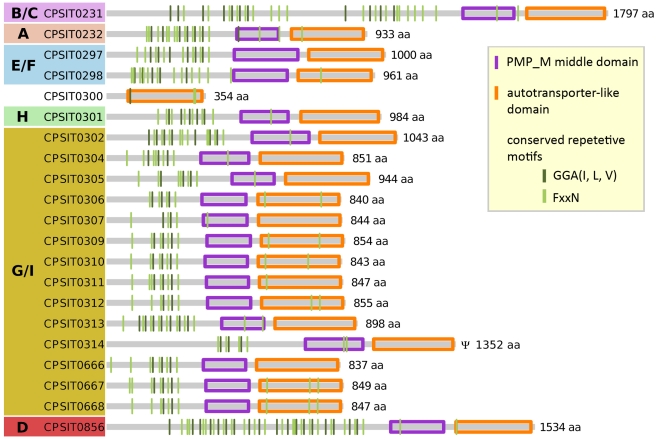
Structure of the pmp-family proteins of *C. psittaci* 6BC. Proteins are ordered by their position in the genome. The letter codes A to G/I indicate the pmp protein subfamilies as previously assigned by [44]. CPSIT_0314 has been reconstructed *in silico*. CPSIT_0300 is a gene remnant.

**Figure 6:**
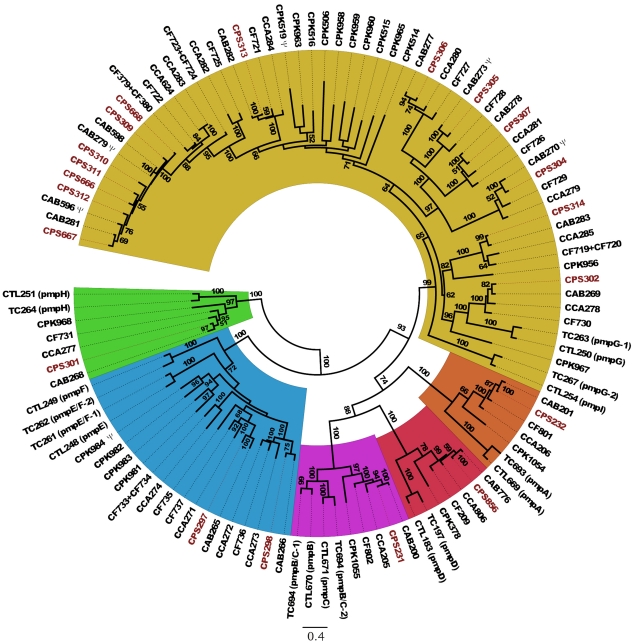
Phylogenetic relationship of chlamydial pmp-family proteins. The maximum-likelihood tree is based on alignments of the conserved PMP_M middle domain and autotransporter domain. Species included in the tree are *C. psittaci* 6BC (CPS), *C. abortus* S26/3 (CAB), *C. felis* Fe/C-56 (CF) , *C. caviae* GPIC (CCA), *C. pneumoniae* LPCoLN (CPK), *C. muridarum* Nigg (TC) and *C. trachomatis* L2/434/Bu (CTL). Pmps cluster into 6 major subfamilies previously designated A (orange), B/C (purple), D (red), E/F (blue), G/I (yellow), and H (green) [44]. Bootstrap values are displayed at the branches. Pseudogenes are marked by 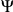.

**Figure 7:**
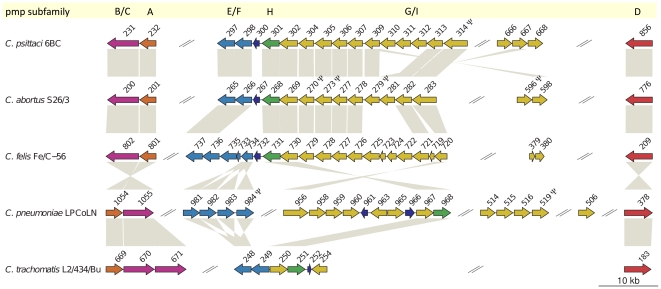
Organization of the pmp-family proteins compared between *C. psittaci* 6BC, *C. abortus* S26/3, *C. felis* Fe/C-56, *C. pneumoniae* LPCoLN, and *C. trachomatis* L2/434/Bu. Arrows indicate the gene orientation. The colour code denotes the membership to a pmp-subfamily. CDSs are designated by the numeric part of the published locus tags. Orthologous genes as inferred from the phylogenetic analysis shown in Figure 6, are connected by grey bars. Pseudogenes are marked by 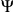.

The second major source of diversity among chlamydial genomes is the group of polymorphic membrane proteins. The pmps are a large protein family likely unique to the *Chlamydiae* [12], [13]. Pmps are characterized by an unusually high level of mutational change within and across species, suggesting relatively fast evolutionary rates and high selective pressures potentially associated with adaptation to different hosts or immune responses [34], [44], [45]. They are present in varying numbers ranging from nine in *C. trachomatis* and *C. muridarum* [46] to 21 putative CDSs in *C. pneumoniae* [34], [47] and *C. psittaci* (this study; Figure 5). The pmps group phylogenetically into six basic subfamilies (A, B/C, D, E/F, G/I, and H; Figure 6; [46]). Of these subfamilies, family G/I is the largest and the most rapidly evolving with numerous evolutionary recent independent events of gene duplication and loss in the various chlamydial lineages (Figure 6). The tendency to a proliferation of G/I family pmps is especially pronounced among the species belonging to the former genus *Chlamydophila* (i.e. the *psittaci*-group, *C. pneumoniae*, and *C. pecorum*). While there are only two G/I pmps present in *C. trachomatis* and *C. muridarum*, there are 14 pmp G/I family genes present in the *C. psittaci* genome (Figure 7).

In *C. psittaci* 6BC, one pmp gene is predicted to be truncated on the N-terminal side (CPSIT_0314). Similar to other *Chlamydiaceae*, a number of pmps harbour long poly-G tracts. Interestingly, while these poly-G stretches appeared to be in frame in the sequence generated by us, in a parallel sequencing effort on *C. psittaci* 6BC [21] the three pmp genes corresponding to CPSIT_0305, CPSIT_0312, and CPSIT_0666 are found to contain frameshifts in these long homopolymeric tracts. Whether this is a sequencing artefact or represents rapid change due to slippage mutations is unclear.

Despite their overall low amino acid and nucleotide similarities, all pmps share a unique domain structure. They contain a C-terminal autotransporter-like domain, a central pmp middle domain and a varying number of the *Chlamydia*-specific short tetrapeptide motifs GGA(I, L, V) and FxxN on the N-terminal side [14], [44], [48] (compare Figure 5). In *C. psittaci* the numbers of conserved tetrapeptide motifs range from two to 18 for GGA(I, L, V), and from four up to 23 for FxxN. On average 9 FxxN and 4.8 GGA(I, L, V) motifs are found per pmp gene. These numbers are similar to other chlamydial species: *C. trachomatis* (13.6 and 6.5) and *C. pneumoniae* (11.3 and 5.0) [44]. Importantly, it has recently been shown that at least two copies of these repetitive tetrapeptide motives are essential for chlamydial adhesion to the host cell [49].

### Putative type III secreted effector proteins

Like a variety of other Gram-negative pathogens, *C. psittaci* uses a conserved type III secretion machinery as a basic mechanism of virulence determination, that allows transporting specific proteins known as type III secreted effectors (T3SE) into the cytoplasm of their host cells [17], [50], [51]. Many of the chlamydial T3S effectors are targeted to the inclusion membrane that encapsulates the pathogen inside their host cell [52]. Hence, these effectors are thought to play a crucial role for the modification of the chlamydial environment and for the survival of *Chlamydiae* in their inclusion vacuole [15].

To predict potential T3S effector proteins in the genomes of *C. psittaci* 6BC, *C. abortus* S26/3, *C. felis* Fe/C-56, *C. caviae* GPIC, *C. pneumoniae* LPCoLN, and *C. trachomatis* L2/434/Bu we used a support vector machine (SVM) classifier developed by Wang et al. [18] that is based on T3S-specific features extracted from the N-terminal amino acid composition profile of proteins and the prediction software EffectiveT3 [19] (see Methods section).

*Chlamydia psittaci* CDSs predicted by these methodologies to encode T3S effector proteins and their orthologs in the other investigated species are presented in Table 3 (and Tables S1, S2, S3, S4, S5, S6, S7). Using a decision threshold of 0.5, 40 CDSs are classified as T3S effectors by the SVM algorithm (Table 3) and 68 CDSs are identified by EffectiveT3 (Table S7). 15 CDSs are identified by both approaches (Table 3).

As can be expected, many of the proteins classified as T3S effectors in *C. psittaci* are homologs to experimentally verified effector proteins from other species. CPSIT_0192, for instance, is orthologous to the important *C. trachomatis* T3S effector Tarp (translocated actin-recruiting protein) [53]. Tarp orthologs are present in all examined chlamydial species (Table 3). CPSIT_0192 has 100% query coverage and 91% sequence identity to *C. abortus* CAB167 and possesses three *Chlamydia*-specific domains of unknown function (DUF1547) and an actin-binding I/LWEQ domain. Query coverage and sequence identity to *C. trachomatis* Tarp CTL0716 are 62% and 37%, respectively. This highlights the high degree of variability in these genes among the *Chlamydiaceae*. Even within *C. trachomatis* variation in the Tarp sequence has been reported [43]. Despite significant sequence differences to *C. trachomatis*, both, the *C. psittaci* and the *C. trachomatis* Tarp are expressed late in the developmental cycle and may have the same function [54], [55].

An important family of T3S effectors tightly associated with the inclusion membrane are the Inc proteins. Members of this family show little general sequence similarity, but share a conspicuous bilobed hydrophobic domain of 60–80 amino acid residues [56]. An enrichment for coiled-coil regions typical for eukaryotic organisms has recently been described for putative Incs [12].

The *C. trachomatis* genome contains seven characterized Inc proteins (Inc A to G). The high sequence diversity in the Inc protein family makes the occurrence of most Inc proteins largely strain-specific. Thus, in *C. psittaci*, of three characterized Inc proteins A, B, and C only Inc B (CPSIT_0532) is conserved enough to be identified as an ortholog to *C. trachomatis* Inc B (CTL0484) by reciprocal BLAST.

In *C. psittaci* both Inc A and B were classified as T3S effectors by both prediction approaches (Table 3). For both proteins, type III secretion has also been experimentally confirmed in *C. psittaci* and *C. pneumoniae* [54], [57]. Based on high immunological activity Inc A was the first Inc protein identified [58]. Inc B modulates host immune responses and might be involved in inclusion development and prevention of early lysosomal fusion [59]. The *C. psittaci* Inc C (CPSIT_0531) has not been classified as a T3SE by the SVM approach (but it is recognized as a T3SE by EffectiveT3; Table S7). This is likely explained by the complete lack of sequence homology in the N-terminal region with respect to the *C. trachomatis* Inc C (CTL0485; query coverage 53%, sequence identity 50%), which is an experimentally verified T3S effector.

Another cluster of genes putatively belonging to the larger Inc protein family and presumably playing similar roles, are the transmembrane head proteins (TMH) [27]. Transmembrane head proteins are characterized by a paired N-terminal transmembrane domain (IncA) followed by alpha-helical coiled-coil domains and show levels of sequence similarity significantly lower than the genome average [27].

In *C. psittaci* the *tmh* locus encodes 8 CDSs (CPSIT_0841, CPSIT_0842, CPSIT_0843, CPSIT_0844, CPSIT_0846, CPSIT_0848, CPSIT_0850, CPSIT_0851), all of which harbour an N-terminal IncA domain. The TMH proteins CPSIT_0844 and CPSIT_0846 were classified as possible T3S effectors by both prediction approaches and are orthologous to *C. abortus* CAB764 and CAB766, respectively (Table 3)

The comparison with *C. felis* and *C. caviae* suggest that the genes encoding the above proteins have arisen from a duplication event in the common ancestor of *C. psittaci* and *C. abortus*. The feline ortholog CF0218 was shown to be distributed throughout the chlamydial inclusion bodies and confirmed to be immunogenic [60], but has not been classified as a T3S effector by our approach.

Besides a number of experimentally confirmed T3S effectors, some proteins with functional annotations that suggest a role in host-pathogen interactions and/or pathogenicity have been classified as T3S effectors. Among this group are a number of genes belonging to the pmp G family (CPSIT_0313, CPSIT_0314 [predicted by SVM], CPSIT_0311, CPSIT_0312, CPSIT_0316 [predicted by EffectiveT3]) and four of the 16 genes located in the plasticity zone. Thus, the adherence factor (CPSIT_0606) located in the PZ (Figure 3) is predicted as T3SE with a high SVM score and by EffectiveT3. Although the adherence factor has orthologs in *C. caviae* GPIC, *C. abortus* S26/3 (only a small gene remnant showing 2% query coverage, but 93% sequence identity) and *C. felis* Fe/C-56 (Table 3), the adherence factor is only predicted to be type III secreted for *C. psittaci* and *C. caviae* orthologs. The adherence factors of *C. felis* (CF0442) and *C. caviae* (CCA_00558) show in comparison to the psittacine adherence factor a query coverage of 90%, and a DNA sequence identity of 45% and 44%, respectively, suggesting a high evolutionary turn over.

### Selection pressure on chlamydial genomes

The basic measure of selective pressure acting on protein coding sequences is the 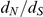-ratio. Generally, low values of 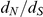 (i.e., values <1) are indicative of purifying selection acting on a given protein coding gene, while values >1 are usually interpreted as evidence for positive selection. Theoretically, the strength of purifying selection depends on the effective population size and the specific mutation and recombination rates of the compared lineages. Smaller effective population sizes and less recombination will lead to relatively larger 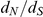-values [61].

**Figure 8:**
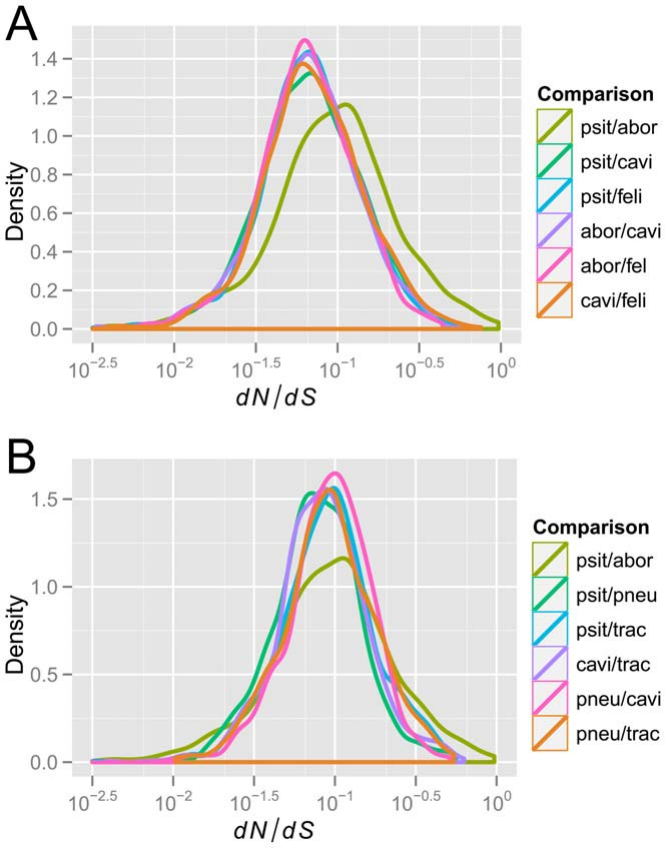
Distributions of 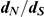 for orthologous genes from pairs of chlamydial genomes. **A.** Distributions for comparisons among the closely related species *C. psittaci*, *C. abortus*, *C. felis*, and *C. caviae*. **B.** Distributions for comparisons among the more distantly related species *C. psittaci* and *C. caviae* vs. *C. pneumoniae* and *C. trachomatis*, respectively. As a point of reference the comparison *C. psittaci* vs. *C. abortus* is included in both plots. Probability density curves were estimated by Gaussian-kernel smoothing.

**Figure 9:**
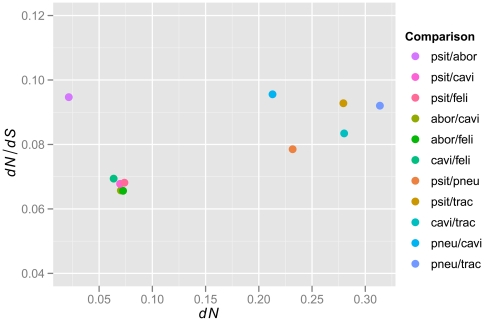
Dependence of median 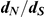 on the genetic distance between genomes estimated as the median non-synonymous substitution rate, 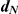.

To characterize differences in selective pressure among chlamydial lineages on a genome-wide scale, we constructed the distributions of 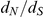 for pairs of chlamydial species over their respective sets of orthologous genes. In agreement with previous findings [62], [63], the shapes of these distributions were highly similar and best fitted by a log-normal distribution (Figure 8). Median 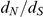-values fell in the range between 0.06 and 0.1 (Figure 9) and are indicative of the strong evolutionary constraints typical for the compact genomes of prokaryotes [63].

It has been observed that 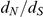 correlates negatively with evolutionary distance, i.e. the smaller the distance between genomes the larger the estimates of 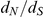, thus leading to an overestimation of positive selection [64]. Such a pattern is not apparent among the chlamydial lineages compared here (Figure 9). In fact, with the notable exception of *C. psittaci* vs. *C. abortus*, pairwise comparisons between the closely related lineages within the “*C. psittaci*-group” show 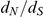-distributions shifted towards lower values, indicative of higher levels genomic conservation, than comparisons across larger genomic distances (compare Figures 8A vs. 8B and Figure 9). In addition to exhibiting less genomic constraint than other comparisons within the same group of lineages, the *C. psittaci*/*C. abortus* comparison also shows the highest overall variance in 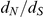-ratios (interquartile range = 0.106; interquartile ranges for all other comparisons range from 0.057 to 0.080)

The median 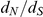-values for pairwise comparisons among chlamydial lineages range between 0.066 and 0.096, and fall thus in the upper third of the range for global median 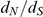-values typically reported for prokaryotes (0.01–0.1 [63]). This is in line with a trend that the weakest purifying selection pressures are seen in obligate parasites, and is probably explained by their relatively small effective population sizes, frequent bottlenecks and low recombination rates [65].

Although no clear phenotypic correlates are apparent, the variability in purifying selection pressure affecting the evolution of different chlamydial lineages may thus also be a reflection of differences in their effective population sizes and/or the frequency of bottlenecks associated with differences in their life styles (e.g. host preferences or differences in pathogenicity may influence the numbers of infected carriers and thus the effective populations sizes of the pathogens).

Using an (arbitrary) cut-off value of a gene-wide 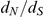-ratio greater than 0.75 for at least one of the nucleotide substitution models, the *C. psittaci*/*C. abortus* comparison is the only one to give a list of potential candidate genes under positive selection (Table 4).

**Table 4:** Genes potentially under positive selection between *C. psittaci* and *C. abortus*.

CCCL CDS1	CDS2	mean 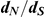 ratio	product description
CPSIT_0350	CAB314	0.974233	putative serine-rich exported protein
CPSIT_0844	CAB764	0.957362	putative TMH-family/IncA-family protein
CPSIT_0161	CAB139	0.911909	putative lipoprotein
CPSIT_0604	CAB549	0.904697	conserved hypothetical protein (plasticity zone)
CPSIT_0469	CAB416	0.821031	putative exported protein
CPSIT_0036	CAB032	0.741458	conserved hypothetical protein
CPSIT_0034	CAB030	0.697462	conserved hypothetical protein
CPSIT_0336	CAB302	0.680659	conserved hypothetical protein
CPSIT_0390	CAB351	0.671977	putative inner membrane protein
CPSIT_0876	CAB793	0.669905	hypothetical protein
CPSIT_0841	CAB760	0.598583	putative TMH-family/IncA-family protein
CPSIT_0685	CAB614	0.595976	co-chaperonin GroES
CPSIT_0545	CAB491	0.582204	hypothetical protein
CPSIT_0207	CAB180	0.537288	small cystein-rich outer membrane protein
CPSIT_0513	CAB460	0.484486	putative lipoprotein

### Metabolic pathways

The *C. psittaci* 6BC genome encodes for all central metabolic pathways such as the glycolytic pathway and the tricarboxylic acid (TCA) cycle. The TCA cycle of obligate intracellular pathogens varies from complete, in e.g. *Coxiella burnetii* [66] and *Rickettsia prowazekii* [67] to absent, in e.g. *Mycoplasma* [68]. Like all other chlamydial species [69], *C. psittaci* 6BC lacks a number of core components of the tricarboxylic acid cycle, namely citrate synthase, aconitase, and isocitrate dehydrogenase. How *Chlamydiaceae* compensate for these deficiencies is not clear.

*Chlamydiaceae* also vary in the completeness of the biotin pathway. Like *C. abortus* S26/3, *C. felis* Fe/C56, and *C. pneumoniae* LPCoLN, the *C. psittaci* 6BC genome contains all genes necessary for the production of biotin from pimeloyl-CoA (Figure S1). In contrast, *C. trachomatis* L2/434/Bu has lost several genes from this pathway such as adenosylmethionine-8-amino-7-oxononanoate aminotransferase *bioA*, dethiobiotin synthetase *bioD*, and biotin synthase *bioB* (Figure S1). Also *C. muridarum* and *C. caviae* exhibit an incomplete biotin gene cluster [27].

Biotin is an essential cofactor involved in many pathways [70]. The phylogenetic distribution of the deficiencies in the biotin biosynthesis pathway within *Chlamydiaceae* suggests at least two independent events of gene loss (one in the common ancestor of *C. trachomatis* and *C. muridarum*, and one in *C. caviae*). This correlates with differences in host specificity. While for *C. trachomatis*, *C. muridarum*, and *C. caviae* only one (or, in the case of *C. muridarum* two closely related) host species has been reported, the host range is markedly broader for the other species [71]. Intracellular organisms generally are prone to loss of function of metabolic genes due to a relaxation of selective constraints in their metabolite-rich environment [72]. This trend may, however, be exacerbated if a restricted host range leads to a reduced effective population size, and thus to a less efficient selection against deleterious mutations.

Differences between the chlamydial species also exist in the purine and pyrimidine pathways (Figures S2 and S3). The genome of *C. psittaci* 6BC contains a gene cluster consisting of IMP dehydrogenase *guaB*, GMP synthase *guaA*, and adenosine deaminase *add* relevant for purine interconversion (Figure S2). These genes are also present in *C. felis*, *C. caviae*, *C. pneumoniae* AR39 (with a *guaB* pseudogene, however), and *C. muridarum* [24], but lack from *C. abortus* (only a *guaB* pseudogene), *C. pneumoniae* LPCoLN, and *C. trachomatis* L2/434/Bu [27], [34]. With respect to pyrimidine interconversion, the genomes of *C. psittaci* 6BC and all other *Chlamydiaceae* encode the conversion of UMP to CTP (uridylate kinase *pyrH*, nucleoside diphosphate kinase *ndk*, and CTP synthase *pyrG*, Figure S3). With the exception of *C. trachomatis* L2/434/Bu all *Chlamydiaceae* examined here also encode orotate phosphoribosyltransferase *pyrE* (Figure S3). Only *C. pneumoniae* encodes uridine kinase *udk*, responsible for the conversion of uridine or cytidine into uridine monophosphate or cytidine monophosphate (UMP/CMP). Also these patterns suggest multiple independent events of loss of function, possibly due to a reduction of selective constraints on metabolic genes, but lack any clear correlation to known differences in host adaptation.

Several studies maintain that *Chlamydiaceae* do not import host dNTPs for DNA synthesis, but convert NTPs to dNTPs [73]–[75]. Both *C. psittaci* 6BC and *C. trachomatis* L2 can obtain all NTPs from the host cell [75]–[77]. In *C. trachomatis* nucleoside phosphate transporters Npt1 and Npt2 are present [74], [78]. Npt1 mediates the exchange of host ATP and bacterial ADP, and Npt2 transports NTPs into the bacterium. An ATP/ADP translocase that enables the RBs to supply themselves with ATP from the host cell, has also been reported for *C. psittaci* 6BC [79]. Our genomic data support this finding. The *C. psittaci* 6BC genome encodes for an ATP/ADP translocase (CPSIT_0474) with 79% sequence identity and 93% query coverage compared to *C. trachomatis* Npt1.

Chlamydial species differ, however, in their requirements with respect to the availability of external sources of NTPs or precursors. While all chlamydial species investigated here are able to synthesize CTP from UTP, only *C. psittaci* and *C. felis* can potentially also interconvert ATP and GTP, because only these two species encode a complete *guaAB-add* cluster. In other words, all *Chlamydiaceae* deficient in the *guaAB-add* cluster have to import ATP, GTP, and UTP or precursors from the host cell [78], [80]. *C. psittaci* and *C. felis* crucially only depend on an external source of UTP and either ATP or GTP or the respective precursors.

Uniquely among *Chlamydiaceae*, *C. pneumoniae* possesses a uridine kinase *udk* (EC 2.7.1.48), converting uridine or cytidine to UMP or CMP [34]. This potentially makes *C. pneumoniae* independent from an external source of UTP if it can take up its precursors, i.e. uridine or cytidine.

Interestingly, it has recently been shown, that a cytosolic 5′-nucleotidase can have phosphotransferase activity in addition to hydrolase activity [81], [82]. For the chlamydial isolates examined in this study, a 5′-nucleotidase (EC 3.1.3.5) has been predicted. If the phosphotransferase activity extends to the chlamydial 5′-nucleotidases, it potentially allows a conversion of (deoxy)guanosine, xanthosine, inosine, (deoxy)adenosine, uridine, and cytidine into their respective monophosphates (Figure S2 and S3). If these precursor molecules can be obtained from the host cell the *guaAB-add* cluster is rendered redundant, decreasing the selection pressure to maintain functional copies of these genes. However, whether *Chlamydiaceae* have the ability to obtain NMP precursors from external sources at all is contentious. While Tribby and Moulder [83] asserted that *C. psittaci* Cal10 incorporates adenine, guanine, their (deoxy)ribonucleosides, later studies [75], [76], [84] found that only precursors which the host cell has converted to nucleotides can successfully be incorporated.

### Conclusions

With the sequencing of the genome of *Chlamydia psittaci* the complete genomic sequences for all species but one (*C. suis*) of the *Chlamydiaceae* has become available. The comparative study of these genomes provides important insights into evolutionary history of this group of closely related intracellular pathogens and allows the identification of genomic differences that may account for the observed variation in virulence, pathogenicity, and host specificity among the species. In this study we made use of the *C. psittaci* genome to focus on the most prominent genomic regions outside of the well-conserved chlamydial core genome: the polymorphic membrane proteins, the chlamydial plasticity zone, and the type III secreted effector proteins. We have shown that the genetic differences of *C. psittaci* with respect to other *Chlamydiaceae* includes an array of unique pmp genes of the G/I subfamily, the lack of a tryptophan operon in the plasticity zone (similar to its sister taxon *C. abortus*), the presence of an uninterrupted adherence factor and a MAC/perforin in the plasticity zone, and a number of candidate type III secreted effectors some of which are not present in all *Chlamydiaceae* or have not been classified as T3SEs in all species. In addition, a number of genes with functional annotations indicative of a role in host-pathogen interactions show some indication of recent positive selection after the split of the *C. psittaci* and *C. abortus* lineages. Further investigation of these genes may provide insights in what enables some species to exploit a wide range of hosts while others seem restricted few closely related host species, or whether these genes indeed may account for differences in virulence and pathogenicity.

## Methods

### Chlamydial genomes

The avian *Chlamydia psittaci* isolate 6BC (GenBank accession number CP002549) was sequenced *de novo* by a combination of Roche 454 pyrosequencing, Illumina and Sanger sequencing to, on average, 487-fold sequence coverage, assembled and annotated as described in [85].

So far complete genomic sequences of seven other chlamydial species have been published: *C. trachomatis* [46], *C. muridarum* [15], *C. pecorum* [41], *C. pneumoniae* [15], [47], [86], *C. caviae* [24], *C. abortus* [27], and *C. felis* [87]. For a phylogenetic analysis based on complete genomes, sequences and annotations for the following publicly available chlamydial species were obtained from NCBI: *Chlamydia muridarum* Nigg (GenBank: AE002160), *Chlamydia trachomatis* (14 strains: AM884176, CP000051, FM872308, FM872307, CP002052, CP002054, AE001273, CP001886, CP001890, CP001930, CP001887, CP001889, CP001888, AM884177), *Chlamydia abortus* S26/3 (CR848038), *Chlamydia caviae* GPIC (AE015925), *Chlamydia felis* Fe/C-56 (AP006861), and *Chlamydia pneumoniae* (5 strains: AE002161, AE001363, BA000008, AE009440, CP001713).

Comparative analyses where mostly restricted to the following subset of the above genomes: *C. trachomatis* L2/434/Bu (AM884176), *C. pneumoniae* LPCoLN (CP001713), *C. felis* Fe/C-56, *C. abortus* S26/3, *C. caviae* GPIC, and *C. psittaci* 6BC.

### Comparative analyses of genome content

For the identification of species- and genus-specific orthologous genes, an all-vs.-all comparison of the translated coding sequences (CDSs) of 6 chlamydial genomes (see above) was performed using BLAT v34 [88]. The BLAT-identified bidirectional hits were filtered, keeping only those with an expect score less than 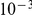 and a cumulative match size of at least one-third of the query sequence length. Where query sequences yielded multiple matches, the match with the highest bit score was retained. Best reciprocal hits by these criteria were considered orthologs for the purpose of this study. A custom R script was written to construct multi-genome match tables and to generate four-way Venn diagrams. Discrepancies from mismatches between putative orthologs in the multi-genome comparison arose in 13 cases and were resolved manually by checking local synteny. To visualize the conservation of genomic context among the *Chlamydiaceae*, a whole-genome synteny plot based on best reciprocal BLAT hits was constructed using the genoPlotR package [89].

Metabolic pathway reconstruction for *C. psittaci* was performed with ASGARD v1.5.3 [90], using the KEGG database [91] as a source for pathway definitions.

For constructing the global phylogeny of the *Chlamydiaceae*, we retrieved a set of 478 orthologous genes conserved across all 24 available chlamydial genomes by all-vs.-all BLAT-comparisons of the CDSs as implemented in the orthology mapping software mercator (http://www.biostat.wisc.edu/cdewey/mercator/). From this set of orthologs, we aligned a random sample of 100 genes with MAFFT v6.717b [92] using the L-INS-i option. The concatenated alignment, spanning 121,258 positions with a total of 58,523 informative sites was employed to reconstruct an unrooted phylogeny by maximum likelihood inference, using PHYML v3.0 [93] under a general-time reversible (GTR) model with six rate categories. To avoid long-branch attraction, intra- and interspecies phylogenies were estimated separately. Base frequencies, transition/transversion ratios, and the gamma distribution parameter 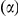 were estimated from the data. Topological robustness was assessed by 100 non-parametric bootstrap replicates.

### Comparative analysis of the polymorphic membrane protein family

Comparative genomics and phylogenetic estimation were used to characterize evolutionary changes affecting the chlamydial polymorphic membrane protein (pmp) family. Predicted pmp sequences were extracted from *C. psittaci* 6BC, *C. abortus* S26/3, *C. caviae* GPIC, *C. felis* Fe/C-56, *C. pneumoniae* LPCoLN, *C. trachomatis* L2/434/Bu, and *C. muridarum* Nigg by searching all translated putative genes for the pmp-specific C-terminal autotransporter 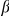-barrel domain and the conserved PMP_M middle domain [27] motifs using the Pfam HMM database. Interrupted pmp genes (in *C. felis*) and annotated pseudogenes (*C. abortus* and *C. pneumoniae*) were reconstructed *in silico* for phylogenetic and comparative analyses.

Due to the inter- and intraspecific divergence of pmp-family proteins and following [27], the phylogenetic analysis of pmp genes was based on alignments of the conserved PMP_M middle domain and the C-terminal autotransporter domain alone. Multiple protein alignments were constructed with MAFFT v6.717b [92] using the L-INS-i option and the BLOSUM80 substitution matrix. A maximum likelihood tree was reconstructed using PHYML [93] under the WAG [94] model of protein evolution. Amino-acid frequencies and the gamma distribution parameter 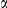 were estimated from the data.

### Test for positive selection and type III secreted proteins

To characterize the nature and strength of selective pressures affecting protein sequences, pairs of orthologous genes between the closely related genomes of *C. psittaci*, *C. abortus*, *C. felis*, and *C. caviae* as well as between the more distantly related genomes of *C. psittaci*, *C. caviae* and *C. pneumoniae*, *C. trachomatis* were identified as bidirectional best hits in an all-against-all BLAT search as described above. Amino acid sequences were aligned using the Needleman-Wunsch global alignment algorithm and the BLOSOM62 substitution matrix as implemented in R, and subsequently translated back to the corresponding DNA sequences. Ratios of the rates of non-synonymous to synonymous nucleotide substitutions per site (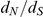), averaged over the entire alignment, were estimated using KaKs_Calculator 2.0 [95]. We calculated 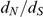-ratios under four of the candidate models of codon substitutions implemented in the software (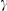-NG, 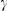-LWL, 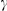-MLWL, and 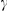-YN), and used 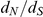-values averaged over all models as an estimate of the selective pressure that affect the compared genomes after their divergence from their most recent common ancestor.

*In silico* prediction of type III secreted (T3S) effector proteins was performed using BPBAac [18]. Briefly, BPBAac uses a Bi-profile Bayes (BPB) approach to feature extraction from training datasets [96] to extract T3S effector features from the position-specific N-terminal amino acid composition (Aac) profile of sets of validated T3S proteins and non-T3S proteins. Bi-Profile Bayes allows representing both the positive and the negative information contained in each peptide sequence in a single posterior probability vector. The posterior probability vectors derived from the training data sets are then used to train a machine learning algorithm, called support vector machine (SVM). Fundamentally, the SVM is a binary classifier that, given two datasets, learns to distinguish between them and to predict the classification of previously unseen samples. The robustness of the classification is expressed by SVM decision values (scores), which indicate the distance in feature space of data points to the nearest point on the decision boundary. Following the practice of [18], we shift the decision threshold from 0 to 0.5 to minimize the number of false positives reported as candidate type III secreted proteins. Additionally, T3S effector proteins were predicted using EffectiveT3 (http://effectors.org) [19]. This approach relies on a taxonomically universal and conserved type III secretion signal sequence in the N-terminus [19]. For prediction we used the standard EffectiveT3 classification module and a cut-off score of 0.9999.

## Supporting Information

Figure S1 **Biotin biosyntesis pathways in** ***C. psittaci*** **6BC.** The genomes of *C. psittaci* 6BC, *C. abortus* S26/3, *C. felis* Fe/C-56, and *C. pneumoniae* LPCoLN encode for all enzymes needed to convert pimeloyl-CoA to biotin. These include 8-amino-7-oxononanoate synthase *bioF*, adenosylmethionine-8-amino-7-oxononanoate aminotransferase *bioA*, dethiobiotin synthetase *bioD*, and biotin synthase *bioB*. The genome of *C. trachomatis* L2/434/Bu encodes only the first step.
(TIF)

Figure S2 **Purine biosynthesis pathway of** ***C. psittaci*** **6BC.** The genomes of *C. psittaci* 6BC and *C. felis* Fe/C-56 encode a guaB/A-add cluster (dehydrogenase *guaB*, GMP synthase *guaA*, adenosine deaminase *add*) for the conversion of AMP, IMP, and GMP, while *C. abortus* S26/3, *C. pneumoniae* LPCoLN, and *C. trachomatis* L2/434/Bu lack this gene cluster. The scheme has been modified after the KEGG PATHWAY database ( www.genome.jp/kegg/pathway.html ). Dashed arrows indicate predicted reaction directions supported by enzyme profiles available from the KEGG ENZYME database ( http://www.genome.jp/kegg/kegg3.html ).
(TIF)

Figure S3 **Pyrimidine biosynthesis pathway of** ***C. psittaci*** **6BC.** (**A**) A scheme of the pyrimidine biosynthesis pathway of *C. psittaci* 6BC, *C. abortus* S26/3, *C. felis* Fe/C56, *C. pneumoniae* LPCoLN, and *C. trachomatis* L2/434/Bu modified after the KEGG PATHWAY database ( www.genome.jp/kegg/pathway.html ). Only *C. pneumoniae* encodes uridine kinase *udk* (EC 2.7.1.48). Only *C. trachomatis* L2/434/Bu lacks orotate phosphoribosyltransferase *pyrE* (EC 2.4.2.10). (**B**) Partial view of the pyrimidine biosynthesis pathway including gene designations: *pyrB*, aspartate carbamoyltransferase; *pyrC*, dihydroorotase; *pyrD*, dihydroorotate dehydrogenase; *pyrE*, orotate phosphoribosyltransferase; *pyrF*, orotidine 5-phosphate decarboxylase; *pyrH*, uridylate kinase; *ndk*, nucleoside diphosphate kinase; *pyrG*, CTP synthase. Dashed arrows indicate predicted reaction directions supported by enzyme profiles available from the KEGG ENZYME database ( http://www.genome.jp/kegg/kegg3.html ).
(TIF)

Table S1 **Predicted type III secreted effectors in** ***Chlamydia psittaci*** **6BC.**
(DOC)

Table S2 **Predicted type III secreted effectors in** ***Chlamydia trachomatis*** **L2/434/Bu.**
(DOC)

Table S3 **Predicted type III secreted effectors in** ***Chlamydia abortus*** **S26/3.**
(DOC)

Table S4 **Predicted type III secreted effectors in** ***Chlamydia felis*** **Fe/C-56.**
(DOC)

Table S5 **Predicted type III secreted effectors in** ***Chlamydia pneumoniae*** **LPCOLN.**
(DOC)

Table S6 **Predicted type III secreted effectors in** ***Chlamydia caviae*** **GPIC.**
(DOC)

Table S7 **Type III secreted effectors in** ***Chlamydia psittaci*** **6BC predicted by EffectiveT3 (** http://www.effectors.org/ **)**
(DOC)
