# Supplementary figures and images for: The Chlamydia psittaci Genome: A Comparative Analysis of Intracellular Pathogens
Source: PLoS One. 2012 Apr 10;7(4):e35097. doi: 10.1371/journal.pone.0035097 (PMC3323650; doi:10.1371/journal.pone.0035097)

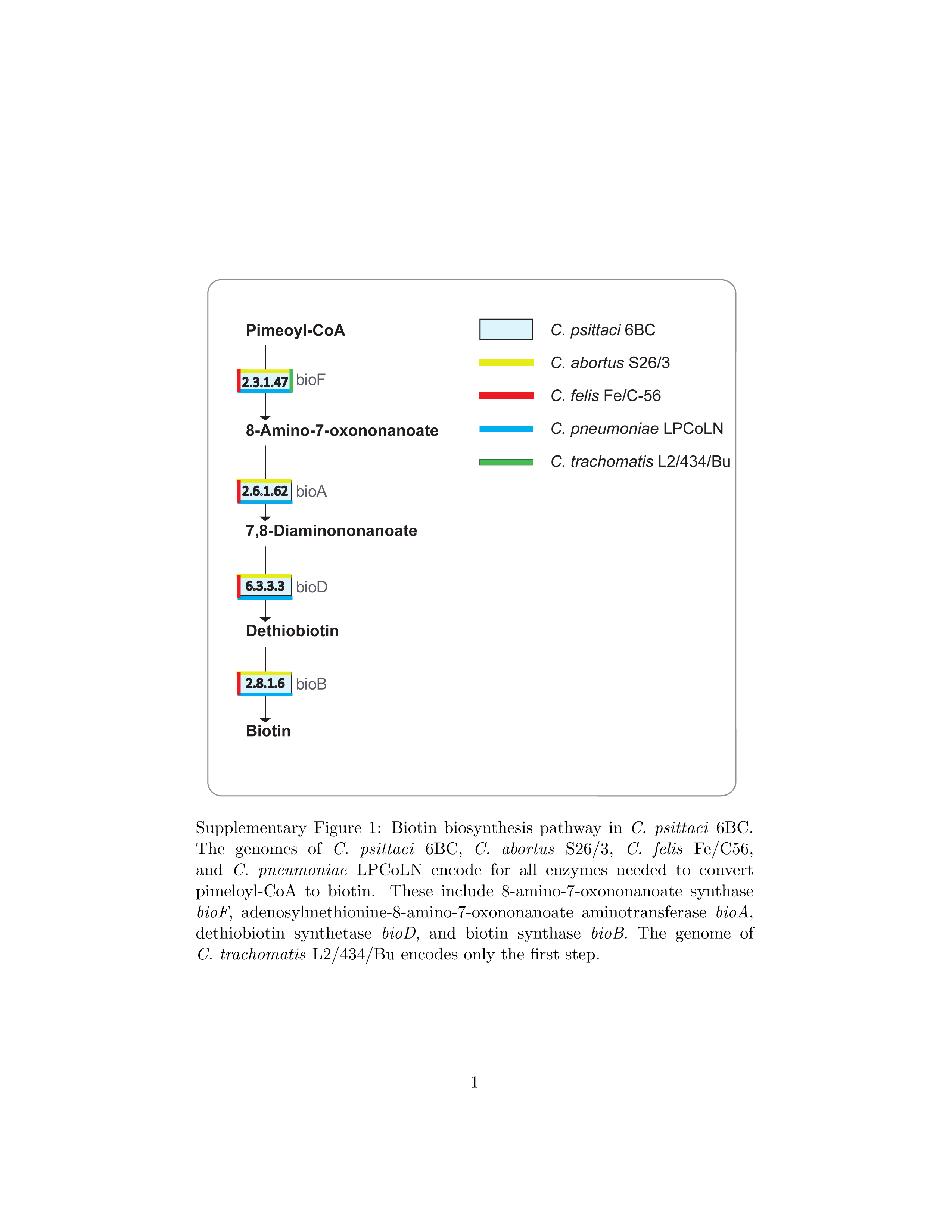

Supplement: Figure S1 — Biotin biosyntesis pathways in C. psittaci 6BC. The genomes of C. psittaci 6BC, C. abortus S26/3, C. felis Fe/C-56, and C. pneumoniae LPCoLN encode for all enzymes needed to convert pimeloyl-CoA to biotin. These include 8-amino-7-oxononanoate synthase bioF, adenosylmethionine-8-amino-7-oxononanoate aminotransferase bioA, dethiobiotin synthetase bioD, and biotin synthase bioB. The genome of C. trachomatis L2/434/Bu encodes only the first step. (TIF) [file pone.0035097.s001.tif]

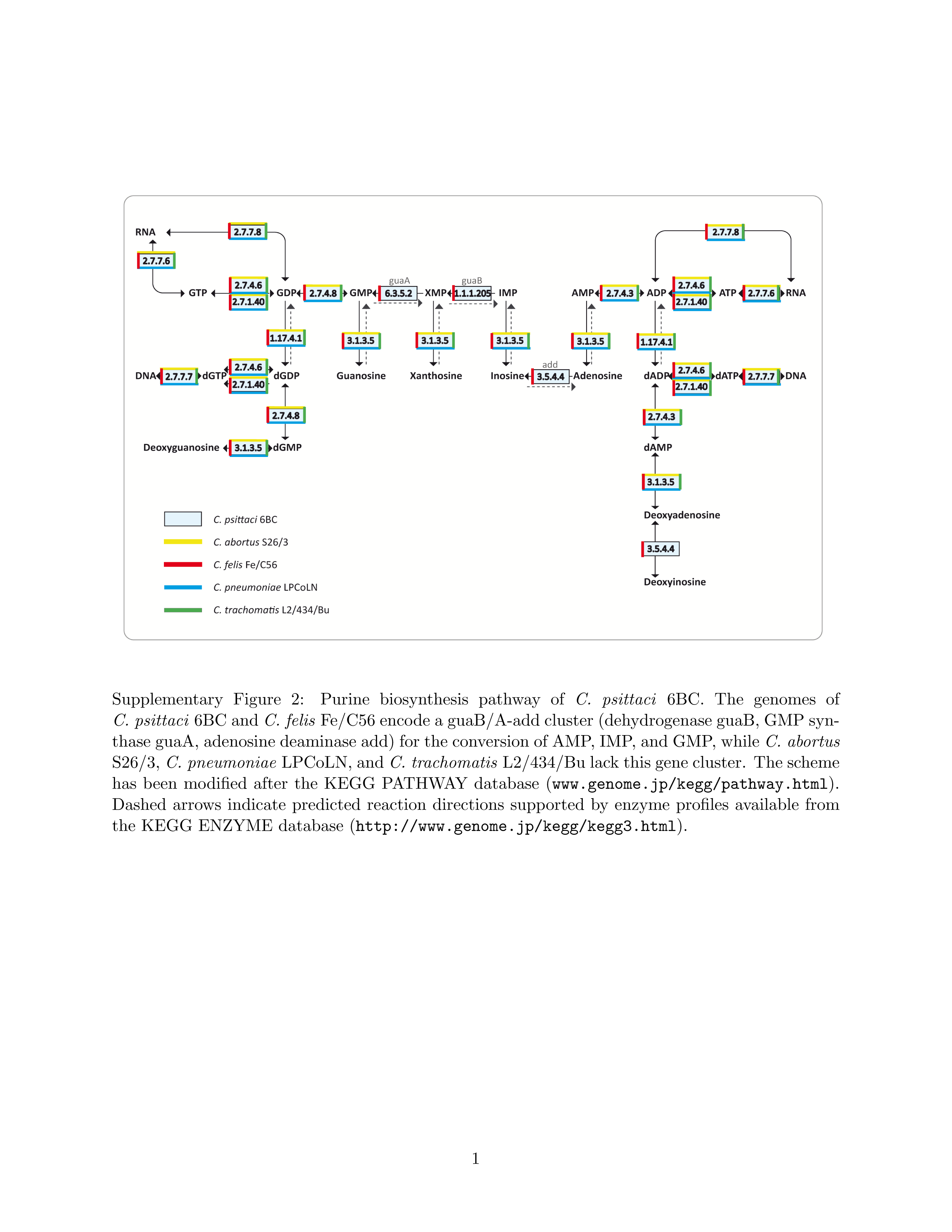

Supplement: Figure S2 — Purine biosynthesis pathway of C. psittaci 6BC. The genomes of C. psittaci 6BC and C. felis Fe/C-56 encode a guaB/A-add cluster (dehydrogenase guaB, GMP synthase guaA, adenosine deaminase add) for the conversion of AMP, IMP, and GMP, while C. abortus S26/3, C. pneumoniae LPCoLN, and C. trachomatis L2/434/Bu lack this gene cluster. The scheme has been modified after the KEGG PATHWAY database ( www.genome.jp/kegg/pathway.html ). Dashed arrows indicate predicted reaction directions supported by enzyme profiles available from the KEGG ENZYME database ( http://www.genome.jp/kegg/kegg3.html ). (TIF) [file pone.0035097.s002.tif]

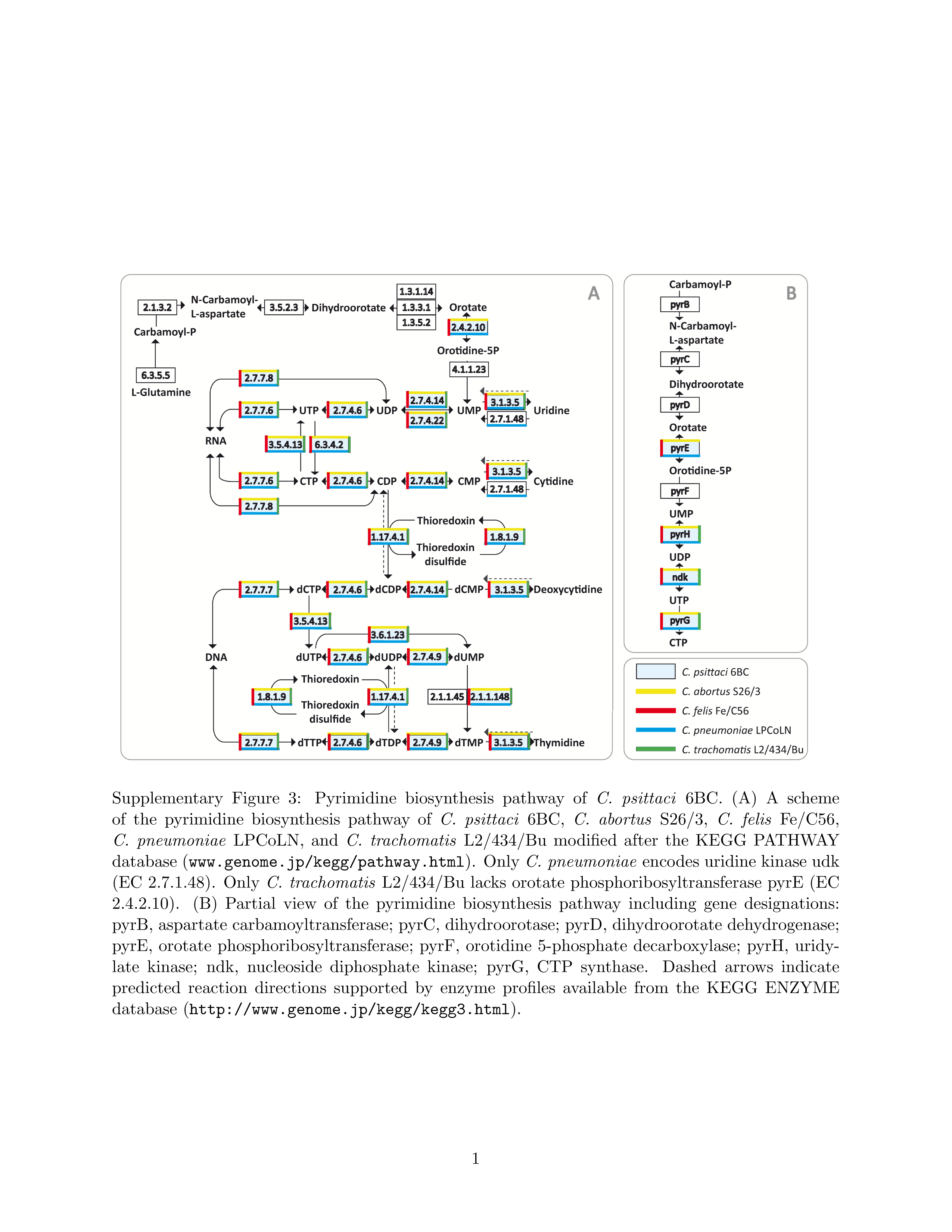

Supplement: Figure S3 — Pyrimidine biosynthesis pathway of C. psittaci 6BC. (A) A scheme of the pyrimidine biosynthesis pathway of C. psittaci 6BC, C. abortus S26/3, C. felis Fe/C56, C. pneumoniae LPCoLN, and C. trachomatis L2/434/Bu modified after the KEGG PATHWAY database ( www.genome.jp/kegg/pathway.html ). Only C. pneumoniae encodes uridine kinase udk (EC 2.7.1.48). Only C. trachomatis L2/434/Bu lacks orotate phosphoribosyltransferase pyrE (EC 2.4.2.10). (B) Partial view of the pyrimidine biosynthesis pathway including gene designations: pyrB, aspartate carbamoyltransferase; pyrC, dihydroorotase; pyrD, dihydroorotate dehydrogenase; pyrE, orotate phosphoribosyltransferase; pyrF, orotidine 5-phosphate decarboxylase; pyrH, uridylate kinase; ndk, nucleoside diphosphate kinase; pyrG, CTP synthase. Dashed arrows indicate predicted reaction directions supported by enzyme profiles available from the KEGG ENZYME database ( http://www.genome.jp/kegg/kegg3.html ). (TIF) [file pone.0035097.s003.tif]
