# Supplementary material for: The Chlamydia psittaci Genome: A Comparative Analysis of Intracellular Pathogens
Source: PLoS One. 2012 Apr 10;7(4):e35097. doi: 10.1371/journal.pone.0035097 (PMC3323650; doi:10.1371/journal.pone.0035097)
Supplement: Table S1 — Predicted type III secreted effectors in Chlamydia psittaci 6BC. (DOC) [file pone.0035097.s004.doc]

**Table S1. Predicted type III secreted effectors in *Chlamydia psittaci* 6BC**

| ORF | SVM value | Annotated |
| --- | --- | --- |
| CPSIT_0357 | 1.957 | hypothetical protein |
| CPSIT_0192 | 1.655 | conserved hypothetical protein / orthologous to C. trachomatis TARP |
| CPSIT_0429 | 1.309 | hypothetical protein |
| CPSIT_0580 | 1.224 | putative inner membrane protein |
| CPSIT_0074 | 1.221 | conserved hypothetical serine rich protein |
| CPSIT_0422 | 1.152 | conserved hypothetical protein |
| CPSIT_0397 | 1.146 | conserved hypothetical protein |
| CPSIT_0463 | 1.109 | putative inner membrane protein |
| CPSIT_0421 | 1.087 | conserved hypothetical protein |
| CPSIT_0532 | 1.087 | inclusion membrane protein B |
| CPSIT_0997 | 1.080 | putative inner membrane protein |
| CPSIT_0606 | 1.061 | adherence factor |
| CPSIT_0245 | 1.054 | carbohydrate isomerase |
| CPSIT_0757 | 1.050 | dihydrodipicolinate reductase |
| CPSIT_0594 | 1.005 | inclusion membrane protein A |
| CPSIT_0220 | 0.971 | 2C-methyl-D-erythritol 2,4-cyclodiphosphate synthase |
| CPSIT_0431 | 0.952 | putative membrane protein |
| CPSIT_0846 | 0.896 | putative TMH-family membrane protein/ incA family protein |
| CPSIT_0844 | 0.864 | putative TMH-family membrane protein/ incA family protein |
| CPSIT_0689 | 0.858 | conserved hypothetical protein |
| CPSIT_0933 | 0.846 | putative membrane protein |
| CPSIT_0314 | 0.829 | polymorphic outer membrane protein G family |
| CPSIT_0749 | 0.769 | conserved hypothetical protein |
| CPSIT_0296 | 0.746 | hypothetical serine rich protein |
| CPSIT_0785 | 0.737 | conserved hypothetical serine rich protein |
| CPSIT_0853 | 0.707 | putative membrane protein |
| CPSIT_0602 | 0.706 | conserved hypothetical protein |
| CPSIT_0962 | 0.704 | Flagellar biosynthesis / type III secretory pathway protein |
| CPSIT_0490 | 0.677 | hypothetical serine rich protein |
| CPSIT_1042 | 0.648 | putative deoxyribonucleotide triphosphate pyrophosphatase / non-conical purine NTP pyrophosphatase, rdgB/HAM1 family |
| CPSIT_0828 | 0.635 | DNA recombination protein/ RmuC domain protein |
| CPSIT_0760 | 0.573 | conserved membrane protein |
| CPSIT_0656 | 0.552 | putative integral membrane protein |
| CPSIT_0974 | 0.549 | trigger factor |
| CPSIT_0313 | 0.545 | polymorphic outer membrane protein G family |
| CPSIT_1054 | 0.540 | 5-formyltetrahydrofolate cyclo-ligase |
| CPSIT_0555 | 0.528 | putative inner membrane protein / putative incA family protein) |
| CPSIT_0930 | 0.517 | putative tRNA(Uracil-5-)-methyltransferase protein |
| CPSIT_0461 | 0.513 | hypothetical protein |
| CPSIT_0767 | 0.506 | 3-phosphoshikimate 1-carboxyvinyltransferase |
| CPSIT_0271 | 0.499 | conserved hypothetical protein |
| CPSIT_0350 | 0.488 | hypothetical protein |
| CPSIT_0316 | 0.487 | polymorphic outer membrane protein G family |
| CPSIT_0249 | 0.477 | uroporphyrinogen-III synthase |
| CPSIT_0139 | 0.445 | replicative DNA helicase |
| CPSIT_0603 | 0.435 | conserved hypothetical protein |
| CPSIT_0813 | 0.426 | conserved hypothetical membrane transport protein |
| CPSIT_0152 | 0.410 | RNA methyltransferase |
| CPSIT_0644 | 0.387 | peptide ABC transporter, ATP-binding protein |
| CPSIT_0008 | 0.359 | putative cell shape-determining protein |
| CPSIT_0649 | 0.346 | diphosphate--fructose-6-phosphate 1-phosphotransferase |
| CPSIT_0247 | 0.341 | conserved hypothetical protein |
| CPSIT_0082 | 0.336 | conserved hypothetical protein |
| CPSIT_0855 | 0.335 | conserved membrane protein |
| CPSIT_1055 | 0.327 | recombinase A |
| CPSIT_0267 | 0.327 | molecular chaperone protein DnaK |
| CPSIT_0335 | 0.319 | 1-deoxy-D-xylulose-5-phosphate synthase |
| CPSIT_0061 | 0.316 | FeS assembly protein SufB |
| CPSIT_0582 | 0.313 | conserved hypothetical protein |
| CPSIT_0295 | 0.310 | putative membrane protein |
| CPSIT_0965 | 0.308 | UTP--glucose-1-phosphate uridylyltransferase |
| CPSIT_0018 | 0.289 | conserved hypothetical protein |
| CPSIT_0068 | 0.284 | putative chromosome partitioning protein |
| CPSIT_0870 | 0.281 | 50S ribosomal protein L25, Ctc-form |
| CPSIT_1011 | 0.279 | hypothetical protein |
| CPSIT_0305 | 0.277 | polymorphic outer membrane protein G family |
| CPSIT_1056 | 0.274 | conserved hypothetical protein |
| CPSIT_0179 | 0.274 | conserved membrane protein |
| CPSIT_0541 | 0.265 | hypothetical protein |
| CPSIT_0434 | 0.265 | cysteine desulfurase |
| CPSIT_0983 | 0.253 | phosphatidylserine decarboxylase |
| CPSIT_0447 | 0.252 | DNA polymerase III subunit delta |
| CPSIT_0901 | 0.241 | hypothetical protein |
| CPSIT_0515 | 0.229 | glycogen phosphorylase |
| CPSIT_0301 | 0.228 | polymorphic outer membrane protein H family |
| CPSIT_0788 | 0.226 | conserved hypothetical protein |
| CPSIT_0491 | 0.225 | conserved hypothetical protein |
| CPSIT_0945 | 0.222 | putative lipoprotein |
| CPSIT_0415 | 0.221 | conserved hypothetical protein |
| CPSIT_0820 | 0.220 | transcription antitermination factor NusB |
| CPSIT_0268 | 0.217 | heat shock protein grpE |
| CPSIT_0019 | 0.215 | conserved hypothetical protein |
| CPSIT_0908 | 0.210 | conserved hypothetical protein |
| CPSIT_0684 | 0.199 | oligoendopeptidase F |
| CPSIT_0274 | 0.188 | conserved hypothetical protein |
| CPSIT_0599 | 0.183 | conserved hypothetical protein |
| CPSIT_0959 | 0.181 | cystein desulfurase |
| CPSIT_0496 | 0.179 | putative type III secretion chaperone |
| CPSIT_0368 | 0.176 | hypothetical protein |
| CPSIT_0953 | 0.170 | cadmium-translocating P-type ATPase |
| CPSIT_0472 | 0.163 | GTP-binding protein |
| CPSIT_0591 | 0.153 | conserved hypothetical protein |
| CPSIT_0990 | 0.149 | lipoyl synthase |
| CPSIT_0320 | 0.146 | aspartyl/glutamyl-tRNA amidotransferase subunit B |
| CPSIT_0755 | 0.143 | aspartate kinase |
| CPSIT_0593 | 0.137 | methyltransferase |
| CPSIT_1012 | 0.121 | outer protein D1 |
| CPSIT_0618 | 0.120 | conserved hypothetical protein |
| CPSIT_0924 | 0.119 | transcription-repair coupling factor |
| CPSIT_0926 | 0.118 | oxygen-independent coproporphyrinogen III oxidase |
| CPSIT_0382 | 0.118 | conserved hypothetical protein |
| CPSIT_0579 | 0.117 | adenylate kinase |
| CPSIT_0685 | 0.098 | co-chaperonin GroES |
| CPSIT_0366 | 0.094 | hypothetical protein |
| CPSIT_0700 | 0.092 | signal recognition particle protein |
| CPSIT_0322 | 0.090 | conserved hypothetical protein |
| CPSIT_0549 | 0.087 | thymidylate kinase |
| CPSIT_0647 | 0.080 | diphosphate--fructose-6-phosphate 1-phosphotransferase |
| CPSIT_0659 | 0.077 | conserved hypothetical protein |
| CPSIT_0174 | 0.076 | conserved hypothetical protein |
| CPSIT_0797 | 0.076 | putative marC family integral membrane protein |
| CPSIT_1036 | 0.074 | lysM domain protein |
| CPSIT_0970 | 0.073 | phosphoenolpyruvate carboxykinase |
| CPSIT_0575 | 0.071 | conserved hypothetical protein |
| CPSIT_0994 | 0.070 | secretion and cellular translocation protein R |
| CPSIT_0186 | 0.062 | putative phosphohydrolase |
| CPSIT_0976 | 0.062 | ATP-dependent Clp protease proteolytic subunit |
| CPSIT_0075 | 0.051 | conserved hypothetical protein |
| CPSIT_0605 | 0.045 | hypothetical protein |
| CPSIT_0175 | 0.044 | conserved hypothetical protein |
| CPSIT_0371 | 0.043 | ribonuclease III |
| CPSIT_0501 | 0.043 | riboflavin biosynthesis protein |
| CPSIT_0297 | 0.041 | polymorphic outer membrane protein E family |
| CPSIT_0458 | 0.039 | 4-hydroxy-3-methylbut-2-en-1-yl diphosphate synthase |
| CPSIT_0294 | 0.035 | 1,4-alpha-glucan branching enzyme |
| CPSIT_0570 | 0.034 | hypothetical protein |
| CPSIT_0666 | 0.029 | polymorphic outer membrane protein G family |
| CPSIT_0589 | 0.025 | ABC transporter ATP-binding protein |
| CPSIT_0787 | 0.015 | conserved membrane protein |
| CPSIT_0180 | 0.007 | putative 2-component regulatory system-sensor histidine kinase |
| CPSIT_0070 | 0.006 | putative peptide ABC transport ATP-binding protein |
| CPSIT_0196 | 0.006 | cytidylate kinase |
| CPSIT_0832 | 0.005 | succinyl-CoA synthase subunit alpha |
| CPSIT_0730 | 0.004 | hypothetical protein |
| CPSIT_0941 | 0.004 | putative metal-dependent hydrolase |
| CPSIT_0237 | 0.000 | putative dnK suppressor protein |
