# Supplementary material for: The Chlamydia psittaci Genome: A Comparative Analysis of Intracellular Pathogens
Source: PLoS One. 2012 Apr 10;7(4):e35097. doi: 10.1371/journal.pone.0035097 (PMC3323650; doi:10.1371/journal.pone.0035097)
Supplement: Table S2 — Predicted type III secreted effectors in Chlamydia trachomatis L2/434/Bu. (DOC) [file pone.0035097.s005.doc]

**Table S2. Predicted type III secreted effectors in *Chlamydia trachomatis* L2/434/Bu**

| ORF | SVM value | Annotated |
| --- | --- | --- |
| CTL0716 | 2.825 | Translocated actin-recruiting phosphoprotein |
| CTL0485 | 1.547 | inclusion membrane protein C |
| CTL0886 | 1.512 | conserved hypothetical protein |
| CTL0373 | 1.350 | inclusion membrane protein G |
| CTL0360 | 1.261 | conserved hypothetical protein |
| CTL0338 | 1.174 | conserved hypothetical protein |
| CTL0117 | 1.164 | transcription-repair coupling factor |
| CTL0841 | 1.103 | putative type III secretion system membrane protein |
| CTL0484 | 1.040 | inclusion membrane protein B |
| CTL0255 | 1.019 | conserved hypothetical protein |
| CTL0096 | 0.995 | cation transporting ATPase |
| CTL0236 | 0.995 | putative type III secretion translocator |
| CTL0411 | 0.991 | phosphatidylcholine-hydrolyzing phospholipase D (PLD) protein |
| CTL0372 | 0.951 | inclusion membrane protein F |
| CTL0612 | 0.915 | putative inner membrane protein (pseudogene) |
| CTL0344 | 0.915 | low calcium response protein E (TTSS effector protein) |
| CTL0744 | 0.891 | putative membrane protein |
| CTL0648 | 0.883 | conserved hypothetical protein |
| CTL0063 | 0.844 | conserved hypothetical protein |
| CTL0452 | 0.842 | oligopeptide transport system membrane permease |
| CTL0611A | 0.797 | candidate inclusion membrane protein |
| CTL0514 | 0.737 | conserved hypothetical protein |
| CTL0443 | 0.732 | conserved hypothetical protein |
| CTL0480 | 0.723 | candidate inclusion membrane protein |
| CTL0730 | 0.717 | DNA repair protein |
| CTL0828 | 0.714 | putative membrane protein |
| CTL0409 | 0.711 | phosphatidylcholine-hydrolyzing phospholipase D (PLD) protein (pseudogene) |
| CTL0064 | 0.670 | conserved hypothetical protein |
| CTL0882 | 0.668 | putative membrane protein |
| CTL0413 | 0.664 | phosphatidylcholine-hydrolyzing phospholipase D (PLD) protein |
| CTL0466 | 0.657 | candidate inclusion membrane protein |
| CTL0639 | 0.654 | putative membrane protein |
| CTL0246 | 0.628 | putative membrane protein |
| CTL0392 | 0.619 | conserved hypothetical protein |
| CTL0534 | 0.595 | glycine cleavage system H protein |
| CTL0713 | 0.580 | 1-acyl-sn-glycerol-3-phosphate acyltransferase |
| CTL0444 | 0.571 | candidate inclusion membrane protein |
| CTL0651 | 0.568 | HSP-70 Cofactor |
| CTL0280 | 0.562 | signal recognition particle, subunit FFH/SRP54 |
| CTL0825 | 0.546 | type III secretion system, membrane protein |
| CTL0247 | 0.528 | putative membrane protein |
| CTL0210 | 0.517 | putative membrane transport protein |
| CTL0115 | 0.485 | coproporphyrinogen oxidase (NAD) |
| CTL0419A | 0.484 | putative membrane protein |
| CTL0759 | 0.479 | replicative DNA helicase |
| CTL0735 | 0.474 | putative membrane protein |
| CTL0393 | 0.472 | microsomal dipeptidase |
| CTL0842 | 0.459 | putative type III secretion system protein |
| CTL0640 | 0.455 | conserved hypothetical protein |
| CTL0221 | 0.449 | conserved hypothetical protein |
| CTL0042 | 0.433 | putative serine/threonine-protein kinase (TTSS effector protein) |
| CTL0885 | 0.416 | conserved hypothetical protein |
| CTL0235 | 0.401 | conserved hypothetical protein |
| CTL0081 | 0.391 | conserved hypothetical protein |
| CTL0320 | 0.386 | GTP-binding protein |
| CTL0090 | 0.385 | Cysteine desulfurase (EC 2.8.1.7) |
| CTL0454 | 0.375 | oligopeptide transport system ATP-binding protein |
| CTL0252 | 0.368 | conserved hypothetical protein |
| CTL0403 | 0.368 | FAD-dependent monooxygenase |
| CTL0124 | 0.361 | 60 kDa chaperonin GroEL |
| CTL0204 | 0.350 | Antitermination protein |
| CTL0275 | 0.347 | signal peptidase I |
| CTL0669 | 0.347 | polymorphic outer membrane protein |
| CTL0137 | 0.342 | conserved hypothetical protein |
| CTL0540 | 0.336 | candidate inclusion membrane protein |
| CTL0557 | 0.333 | V-type sodium ATP synthase subunit I |
| CTL0266 | 0.333 | putative exported protein |
| CTL0525 | 0.323 | conserved hypothetical protein |
| CTL0402 | 0.321 | putative integral membrane protein |
| CTL0880 | 0.317 | putative integral membrane protein |
| CTL0582 | 0.290 | triosephosphate isomerase |
| CTL0652 | 0.290 | chaperone protein |
| CTL0303 | 0.282 | conserved hypothetical protein |
| CTL0014 | 0.281 | conserved hypothetical protein |
| CTL0342 | 0.281 | 4-alpha-glucanotransferase |
| CTL0010 | 0.279 | putative membrane protein |
| CTL0312 | 0.271 | conserved hypothetical protein |
| CTL0043 | 0.259 | Type III secretion structural protein (outer membrane ring) |
| CTL0105 | 0.252 | conserved hyporthetical protein |
| CTL0599 | 0.251 | hypothetical protein |
| CTL0619 | 0.248 | putative integral membrane protein |
| CTL0709 | 0.245 | putative membrane protein |
| CTL0748 | 0.244 | methyltransferase |
| CTL0884 | 0.240 | conserved hypothetical protein |
| CTL0576 | 0.238 | hypothetical protein |
| CTL0821 | 0.234 | lipoic acid synthetase |
| CTL0371 | 0.228 | inclusion membrane protein E |
| CTL0259 | 0.216 | aspartyl/glutamyl-tRNA(Asn/Gln) amidotransferase subunit B |
| CTL0588 | 0.208 | DNA polymerase III subunit gamma/tau |
| CTL0791 | 0.194 | putative membrane protein |
| CTL0883 | 0.190 | conserved hypothetical protein |
| CTL0475 | 0.189 | candidate inclusion membrane protein |
| CTL0079 | 0.184 | phosphoenolpyruvate carboxykinase (GTP) |
| CTL0135 | 0.182 | tRNA delta(2)-isopentenylpyrophosphate transferase |
| CTL0629 | 0.182 | putative oxidoreductase |
| CTL0765 | 0.174 | conserved hypothetical protein |
| CTL0699 | 0.172 | putative membrane protein |
| CTL0056 | 0.169 | cysteine desulfurase |
| CTL0894 | 0.167 | transcriptional regulatory protein |
| CTL0338A | 0.157 | conserved hypothetical protein |
| CTL0238 | 0.156 | conserved hypothetical protein |
| CTL0671 | 0.151 | polymorphic outer membrane protein |
| CTL0385 | 0.151 | ABC transporter, ATP-binding component |
| CTL0604 | 0.150 | putative lipoprotein |
| CTL0667 | 0.145 | polyA polymerase |
| CTL0763 | 0.138 | holliday junction DNA helicase |
| CTL0623 | 0.137 | 3-dehydroquinate synthase |
| CTL0549 | 0.136 | ribonuclease III |
| CTL0500 | 0.135 | glycogen phosphorylase |
| CTL0459 | 0.134 | pyrophosphate--fructose 6-phosphate 1-phosphotransferase |
| CTL0335 | 0.127 | putative integral membrane protein |
| CTL0647 | 0.124 | ABC transporter substrate-binding component |
| CTL0407 | 0.122 | lipoprotein release ATP-binding component |
| CTL0481 | 0.119 | candidate inclusion membrane protein |
| CTL0059 | 0.093 | ABC transport protein, ATPase component |
| CTL0491 | 0.090 | 3-oxoacyl-(acyl-carrier-protein) synthase III |
| CTL0712 | 0.088 | cytidylate kinase |
| CTL0802 | 0.088 | putatve rRNA methylase (SpoU family) |
| CTL0305 | 0.087 | conserved hypothetical protein |
| CTL0125 | 0.080 | UDP-N-acetylmuramoyl-tripeptide--D-alanyl-D-alanine ligase |
| CTL0202 | 0.068 | putative SAM-dependent methyltransferase |
| CTL0065 | 0.065 | conserved hypothetical protein |
| CTL0873 | 0.063 | RNA polymerase sigma-54 factor |
| CTL0451 | 0.061 | oligopeptide transport system membrane permease |
| CTL0394 | 0.059 | oligopeptide transport system binding protein |
| CTL0871 | 0.056 | uracil-DNA glycosylase |
| CTL0839 | 0.055 | type III secretion chaperone (low calcium response protein H) |
| CTL0635 | 0.054 | metal-dependent hydrolase |
| CTL0133 | 0.054 | putative membrane protein |
| CTL0742 | 0.051 | conserved hypothetical protein |
| CTL0306 | 0.050 | conserved hypothetical protein |
| CTL0500A | 0.045 | hypothetical protein |
| CTL0687 | 0.036 | ubiquinone/menaquinone biosynthesis methyltransferase |
| CTL0263 | 0.036 | ribonuclease HIII |
| CTL0815 | 0.034 | putative methyltransferase |
| CTL0527 | 0.033 | Chromosomal replication initiation protein |
| CTL0844 | 0.032 | threonyl-tRNA synthetase |
| CTL0261 | 0.031 | putative membrane protein |
| CTL0851 | 0.030 | sigma regulatory family protein-PP2C phosphatase |
| CTL0503 | 0.029 | inner membrane protein |
| CTL0120 | 0.020 | AMP nucleosidase |
| CTL0550 | 0.017 | DNA repair protein |
| CTL0620 | 0.009 | 3-phosphoshikimate 1-carboxyvinyltransferase |
| CTL0207 | 0.003 | LSU ribosomal protein L20P |
| CTL0561 | 0.002 | conserved hypothetical protein |
