# Supplementary material for: The Chlamydia psittaci Genome: A Comparative Analysis of Intracellular Pathogens
Source: PLoS One. 2012 Apr 10;7(4):e35097. doi: 10.1371/journal.pone.0035097 (PMC3323650; doi:10.1371/journal.pone.0035097)
Supplement: Table S3 — Predicted type III secreted effectors in Chlamydia abortus S26/3. (DOC) [file pone.0035097.s006.doc]

**Table S3. Predicted type III secreted effectors in *Chlamydia abortus* S26/3**

| ORF | SVM value | Annotated |
| --- | --- | --- |
| CAB167 | 1.958 | conserved hypothetical protein |
| CAB876 | 1.459 | conserved hypothetical protein |
| CAB673 | 1.372 | hypothetical protein |
| CAB376 | 1.336 | conserved hypothetical protein |
| CAB708 | 1.334 | conserved membrane protein |
| CAB522 | 1.293 | putative inner membrane protein |
| CAB151 | 1.157 | conserved hypothetical protein |
| CAB706 | 1.110 | conserved hypothetical serine-rich protein |
| CAB617 | 1.094 | hypothetical membrane protein |
| CAB191 | 1.087 | conserved hypothetical protein |
| CAB383A | 1.066 | putative conserved membrane protein (pseudogene) |
| CAB680 | 1.065 | putative dihydrodipicolinate reductase |
| CAB402 | 0.994 | hypothetical protein |
| CAB910 | 0.978 | putative inner membrane protein |
| CAB273 | 0.969 | polymorphic outer membrane protein (pseudogene) |
| CAB410 | 0.890 | putative inner membrane protein |
| CAB437 | 0.841 | hypothetical serine-rich protein |
| CAB255 | 0.823 | putative membrane protein |
| CAB536 | 0.795 | inclusion membrane protein |
| CAB283 | 0.787 | polymorphic outer membrane protein |
| CAB264 | 0.763 | hypothetical serine rich protein |
| CAB545 | 0.746 | putative membrane protein |
| CAB357 | 0.741 | conserved hypothetical protein |
| CAB747 | 0.726 | putative DNA recombination protein |
| CAB887 | 0.720 | trigger factor |
| CAB412 | 0.718 | putative inner membrane protein |
| CAB760 | 0.698 | putative TMH-family membrane protein |
| CAB215 | 0.695 | conserved hypothetical protein |
| CAB500 | 0.651 | putative inner membrane protein |
| CAB594 | 0.621 | conserved hypothetical protein |
| CAB177 | 0.590 | conserved hypothetical protein |
| CAB952 | 0.587 | conserved hypothetical protein |
| CAB241 | 0.574 | conserved hypothetical protein |
| CAB809 | 0.572 | conserved hypothetical protein |
| CAB775 | 0.568 | conserved membrane protein |
| CAB964 | 0.561 | formyltetrahydrofolate cycloligase |
| CAB499 | 0.506 | conserved hypothetical protein |
| CAB764 | 0.500 | putative TMH-family membrane protein |
| CAB237 | 0.496 | heat shock chaperone protein |
| CAB063 | 0.477 | conserved hypothetical (serine rich) protein |
| CAB683 | 0.474 | conserved membrane protein |
| CAB138 | 0.463 | putative membrane protein |
| CAB637 | 0.450 | conserved hypothetical protein |
| CAB282 | 0.441 | polymorphic outer membrane protein |
| CAB923 | 0.402 | putative inner membrane protein |
| CAB843 | 0.399 | putative transcription-repair coupling factor |
| CAB320 | 0.396 | putative membrane protein (pseudogene) |
| CAB360 | 0.381 | conserved hypothetical protein |
| CAB511 | 0.369 | conserved hypothetical protein |
| CAB734 | 0.368 | conserved hypothetical membrane protein |
| CAB903 | 0.367 | lipoic acid synthetase |
| CAB134 | 0.357 | conserved hypothetical protein |
| CAB273 | 0.347 | polymorphic outer membrane protein (pseudogene) |
| CAB301 | 0.346 | 1-deoxy-D-xylulose 5-phosphate synthase |
| CAB399 | 0.345 | putative DNA-binding protein |
| CAB330 | 0.336 | putative inner membrane protein |
| CAB370 | 0.334 | conserved hypothetical protein |
| CAB674 | 0.328 | putative transmembrane protein |
| CAB965 | 0.327 | recombinase A |
| CAB015 | 0.318 | conserved hypothetical protein |
| CAB154 | 0.309 | conserved membrane protein |
| CAB072 | 0.303 | putative lipoprotein |
| CAB849 | 0.302 | putative RNA methyltransferase |
| CAB387 | 0.299 | putative cysteine desulfurase |
| CAB257 | 0.288 | putative inner membrane protein |
| CAB218 | 0.286 | putative membrane protein |
| CAB596 | 0.284 | polymorphic outer membrane protein (pseudogene) |
| CAB690 | 0.277 | 3-phosphoshikimate 1-carboxyvinyltransferase |
| CAB477 | 0.273 | inclusion membrane protein B |
| CAB773 | 0.253 | putative membrane protein |
| CAB583 | 0.234 | pyrophosphate--fructose 6-phosphate 1-phosphotransferase |
| CAB789 | 0.233 | putative 50S ribosomal protein L25 |
| CAB907 | 0.231 | putative type III export protein |
| CAB219 | 0.229 | conserved hypothetical protein |
| CAB448 | 0.222 | riboflavin biosynthesis protein |
| CAB509 | 0.222 | conserved hypothetical protein |
| CAB740 | 0.220 | putative transcription termination-related protein |
| CAB581 | 0.215 | putative pyrophosphate-dependent phosphofructokinase |
| CAB354 | 0.190 | Na%2B-translocating NADH-quinone reductase subunit C |
| CAB548 | 0.185 | hypothetical protein |
| CAB051 | 0.183 | putative ABC transporter |
| CAB182 | 0.182 | putative sulfur-rich membrane protein |
| CAB879 | 0.181 | putative UDP-N-acetylhexosamine pyrophosphorylase |
| CAB419 | 0.170 | GTP-binding protein (Elongation factor) |
| CAB723 | 0.170 | conserved hypothetical protein |
| CAB161 | 0.170 | putative exported protein |
| CAB555 | 0.169 | conserved hypothetical lipoprotein (pseudogene) |
| CAB054 | 0.160 | putative cysteine desulfurase |
| CAB516 | 0.153 | conserved membrane protein (pseudogene) |
| CAB246 | 0.149 | conserved hypothetical protein |
| CAB057 | 0.146 | putative chromosome partitioning protein |
| CAB238 | 0.145 | GrpE protein(hsp-70 cofactor) |
| CAB016 | 0.145 | conserved hypothetical protein |
| CAB289 | 0.138 | putative inner membrane protein |
| CAB064 | 0.136 | conserved hypothetical protein |
| CAB588 | 0.136 | putative transmembrane protein |
| CAB442 | 0.117 | 4-alpha-glucanotransferase |
| CAB438 | 0.093 | conserved hypothetical protein |
| CAB628 | 0.092 | signal recognition particle protein |
| CAB397 | 0.084 | probable aminopeptidase |
| CAB531 | 0.083 | ABC transporter. ATP-binding component |
| CAB709 | 0.080 | conserved hypothetical serine-rich protein |
| CAB828 | 0.079 | conserved hypothetical protein |
| CAB294 | 0.079 | hypothetical protein |
| CAB152 | 0.078 | conserved hypothetical protein |
| CAB884 | 0.075 | putative phosphoenolpyruvate carboxykinase |
| CAB171 | 0.075 | putative cytidylate kinase |
| CAB845 | 0.074 | putative coproporphyrinogen biosynthesis-related protein |
| CAB578 | 0.073 | putative peptide ATP-binding component of ABC transporter |
| CAB293 | 0.071 | hypothetical protein |
| CAB719 | 0.067 | putative transmembrane protein |
| CAB287 | 0.066 | aspartyl/glutamyl-tRNA amidotransferase subunit B |
| CAB270 | 0.060 | polymorphic outer membrane protein (pseudogene) |
| CAB510 | 0.056 | conserved hypothetical protein |
| CAB487 | 0.056 | putative inner membrane protein |
| CAB217 | 0.053 | putative membrane protein |
| CAB974 | 0.053 | putative membrane protein |
| CAB867 | 0.051 | putative cation transport related membrane protein |
| CAB638 | 0.048 | conserved hypothetical exported protein |
| CAB567 | 0.048 | conserved hypothetical protein (pseudogene) |
| CAB924 | 0.047 | putative inner membrane protein |
| CAB495 | 0.045 | thymidylate kinase |
| CAB334 | 0.043 | ribonuclease III |
| CAB262 | 0.035 | 1.4-alpha-glucan branching enzyme |
| CAB279 | 0.030 | polymorphic outer membrane protein (pseudogene) |
| CAB598 | 0.030 | polymorphic outer membrane protein |
| CAB470 | 0.030 | 3-oxoacyl-(acyl-carrier-protein) synthase III |
| CAB211 | 0.029 | putative tetraacyldisaccharide 4'-kinase |
| CAB443 | 0.027 | putative Type III secretion chaperone |
| CAB465 | 0.026 | conserved hypothetical protein |
| CAB331 | 0.019 | putative inner membrane protein |
| CAB751 | 0.017 | succinyl-CoA synthetase alpha chain |
| CAB611 | 0.013 | conserved hypothetical protein |
| CAB059 | 0.011 | putative peptide ABC transport ATP-binding protein |
| CAB489 | 0.010 | putative ABC transport integral membrane subunit |
| CAB678 | 0.004 | putative aspartokinase |
| CAB488 | 0.000 | ABC transporter. ATP-binding component |
| CAB206 | 0.000 | conserved hypothetical protein |
