# Supplementary material for: The Chlamydia psittaci Genome: A Comparative Analysis of Intracellular Pathogens
Source: PLoS One. 2012 Apr 10;7(4):e35097. doi: 10.1371/journal.pone.0035097 (PMC3323650; doi:10.1371/journal.pone.0035097)
Supplement: Table S4 — Predicted type III secreted effectors in Chlamydia felis Fe/C-56. (DOC) [file pone.0035097.s007.doc]

**Table S4. Predicted type III secreted effectors in *Chlamydia felis* Fe/C−56**

| ORF | SVM value | Annotated |
| --- | --- | --- |
| CF0837 | 1.964 | hypothetical protein |
| CF0761 | 1.877 | conserved hypothetical protein |
| CF0618 | 1.734 | hypothetical protein |
| CF0942 | 1.691 | hypothetical protein |
| CF0129 | 1.452 | hypothetical protein |
| CF0277 | 1.234 | conserved hypothetical protein |
| CF0640 | 1.223 | conserved hypothetical protein |
| CF0619 | 1.063 | hypothetical protein |
| CF0300 | 1.054 | hypothetical protein |
| CF0853 | 1.050 | conserved hypothetical protein |
| CF0073 | 0.925 | conserved hypothetical protein |
| CF0719 | 0.924 | outer membrane protein |
| CF0214 | 0.916 | hypothetical protein |
| CF0516 | 0.882 | inclusion membrane protein |
| CF0991 | 0.848 | wall surface anchor family protein |
| CF0353 | 0.845 | signal recognition particle GTPase |
| CF0785 | 0.833 | hypothetical protein |
| CF0738 | 0.831 | hypothetical protein |
| CF0812 | 0.822 | 2-C-methyl-D-erythritol 2,4-cyclodiphosphate synthase |
| CF0678 | 0.795 | hypothetical protein |
| CF0854 | 0.781 | conserved hypothetical protein |
| CF0556 | 0.740 | conserved hypothetical protein |
| CF0485 | 0.719 | conserved hypothetical protein |
| CF0473 | 0.680 | hypothetical protein |
| CF0059 | 0.679 | wall surface anchor family protein |
| CF0305 | 0.672 | aspartokinase III |
| CF0941 | 0.663 | conserved hypothetical protein |
| CF0966 | 0.640 | serine/threonine protein kinase |
| CF0707 | 0.637 | hypothetical protein |
| CF0685 | 0.636 | hypothetical protein |
| CF0219 | 0.616 | hypothetical protein |
| CF0507 | 0.610 | ABC transporter of dipeptides |
| CF0303 | 0.590 | dihydrodipicolinate reductase |
| CF0494 | 0.575 | synaptonemal complex protein |
| CF0172 | 0.575 | conserved hypothetical protein |
| CF0625 | 0.563 | conserved hypothetical protein |
| CF0591 | 0.556 | cell wall associated hydrolases |
| CF0765 | 0.540 | heat shock protein HSP70 cofactor |
| CF0259 | 0.536 | conserved hypothetical protein |
| CF0498 | 0.532 | serine esterase esterase |
| CF0031 | 0.522 | yggv family hypothetical protein |
| CF0095 | 0.512 | FKBP-type peptidyl-prolyl cis-trans isomerase |
| CF0533 | 0.486 | hypothetical protein |
| CF0500 | 0.483 | thymidylate kinase |
| CF0106 | 0.474 | type III secretory flagellar biosynthesis |
| CF0275 | 0.447 | conserved hypothetical protein |
| CF0671 | 0.442 | DNA polymerase III gamma/tau |
| CF0721 | 0.433 | polymorphic outer membrane protein 9 |
| CF0248 | 0.432 | conserved hypothetical protein |
| CF0293 | 0.432 | 3-phosphoshikimate 1-carboxyvinyltransferase |
| CF0764 | 0.405 | heat shock protein HSP70 cofactor |
| CF0646 | 0.404 | hypothetical protein |
| CF0382 | 0.392 | hypothetical protein |
| CF0421 | 0.383 | hypothetical protein |
| CF0611 | 0.381 | hypothetical protein |
| CF0380 | 0.373 | polymorphic outer membrane protein |
| CF0388 | 0.363 | myosin heavy chain major plasmodial |
| CF0274 | 0.354 | conserved hypothetical protein |
| CF0076 | 0.342 | type III secretion flagellar biosynthesis inner membrane protein |
| CF0826 | 0.338 | hypothetical protein |
| CF0954 | 0.337 | ABC transporter |
| CF0115 | 0.336 | cadmium/zinc cation transporting ATPase |
| CF0098 | 0.332 | phosphoenolpyruvate carboxykinase |
| CF0537 | 0.323 | hypothetical protein |
| CF0867 | 0.318 | conserved hypothetical protein |
| CF0018 | 0.297 | mediator of ATP dependent DNA homologous recombination |
| CF0948 | 0.296 | chromosome partitioning protein |
| CF0458 | 0.289 | inclusion membrane protein |
| CF0851 | 0.286 | inclusion membrane protein |
| CF0060 | 0.286 | wall surface anchor family protein |
| CF0778 | 0.274 | tRNA/rRNA methyltransferase |
| CF0084 | 0.271 | branched-chain amino acid transport system carrier protein |
| CF0668 | 0.269 | hypothetical protein |
| CF0258 | 0.257 | conserved hypothetical protein |
| CF0295 | 0.257 | adenosylmethionine-8-amino-7-oxononanoate aminotransferase |
| CF0831 | 0.253 | undecaprenyl pyrophosphate synthetase |
| CF0706 | 0.237 | hypothetical protein |
| CF0311 | 0.235 | conserved hypothetical protein |
| CF0472 | 0.231 | hypothetical protein |
| CF0760 | 0.223 | hypothetical protein |
| CF0234 | 0.218 | DNA recombination protein |
| CF0607 | 0.210 | cysteine desulfurase |
| CF0731 | 0.207 | polymorphic outer membrane protein H family |
| CF0555 | 0.205 | conserved hypothetical protein |
| CF0714 | 0.196 | aspartyl/glutamyl-tRNA amidotransferase subunit B |
| CF0567 | 0.192 | ABC transporter of metals |
| CF0594 | 0.190 | conserved hypothetical protein |
| CF0791 | 0.189 | tetraacyldisaccharide 4'-kinase |
| CF0569 | 0.188 | ABC transporter of metals |
| CF0575 | 0.179 | GTP-binding protein |
| CF0752 | 0.177 | conserved hypothetical protein |
| CF0440 | 0.176 | transcription factor/trp operon repressor |
| CF0330 | 0.172 | V-type ATP synthase subunit I |
| CF0725 | 0.165 | polymorphic outer membrane protein G/9 family |
| CF0934 | 0.164 | conserved hypothetical protein |
| CF0399 | 0.151 | ABC transporter of peptides |
| CF0551 | 0.149 | 4-alpha-glucanotransferase |
| CF0622 | 0.146 | conserved hypothetical protein |
| CF0532 | 0.143 | glycogen phosphorylase |
| CF0379 | 0.143 | polymorphic outer membrane protein |
| CF0508 | 0.141 | hypothetical protein |
| CF0722 | 0.132 | polymorphic outer membrane protein |
| CF0613 | 0.123 | conserved hypothetical protein |
| CF0949 | 0.122 | conserved hypothetical protein |
| CF0217 | 0.119 | myosin heavy chain form B |
| CF0609 | 0.116 | hypothetical protein |
| CF0855 | 0.115 | conserved hypothetical protein |
| CF0109 | 0.115 | cysteine desulfurase |
| CF0418 | 0.111 | hypothetical protein |
| CF0734 | 0.110 | outer membrane protein |
| CF0495 | 0.096 | zinc-dependent dipeptidase |
| CF0973 | 0.081 | hypothetical protein |
| CF0503 | 0.079 | conserved hypothetical protein |
| CF0132 | 0.078 | tRNA/rRNA methyltransferase |
| CF0637 | 0.073 | hypothetical protein |
| CF0833 | 0.072 | cytidylate kinase |
| CF0729 | 0.072 | polymorphic outer membrane protein pmp9 family |
| CF0450 | 0.072 | hypothetical protein |
| CF0871 | 0.068 | tRNA/rRNA methyltransferase |
| CF0788 | 0.066 | sugar-phosphate isomerase-like protein |
| CF0705 | 0.065 | hypothetical protein |
| CF0506 | 0.058 | ABC transporter |
| CF1000 | 0.054 | cell division related rod shape-determining membrane protein |
| CF0137 | 0.052 | oxygen-independent coproporphyrinogen III oxidase |
| CF0194 | 0.043 | 50S ribosomal protein L25 |
| CF0699 | 0.042 | 1-deoxy-D-xylulose 5-phosphate synthase |
| CF0208 | 0.040 | phospholipid synthesis protein |
| CF0584 | 0.038 | 1-hydroxy-2-methyl-2-butenyl 4-diphosphate synthase |
| CF0664 | 0.036 | ribonuclease III |
| CF0019 | 0.030 | formyltetrahydrofolate cycloligase |
| CF0585 | 0.025 | conserved hypothetical protein |
| CF0103 | 0.019 | UDP-N-acetylglucosamine pyrophosphorylase |
| CF0888 | 0.014 | conserved hypothetical protein |
| CF0799 | 0.013 | conserved hypothetical protein |
| CF0422 | 0.008 | hypothetical protein |
| CF0748 | 0.001 | 2-dehydro-3-deoxyphosphooctonate aldolase |
