# Supplementary material for: The Chlamydia psittaci Genome: A Comparative Analysis of Intracellular Pathogens
Source: PLoS One. 2012 Apr 10;7(4):e35097. doi: 10.1371/journal.pone.0035097 (PMC3323650; doi:10.1371/journal.pone.0035097)
Supplement: Table S5 — Predicted type III secreted effectors in Chlamydia pneumoniae LPCOLN. (DOC) [file pone.0035097.s008.doc]

**Table S5. Predicted type III secreted effectors in *Chlamydia pneumoniae* LPCoLN**

| ORF | SVM value | Annotated |
| --- | --- | --- |
| CPK_ORF01089 | 2.073 | conserved hypothetical protein |
| CPK_ORF00890 | 1.597 | putative membrane protein |
| CPK_ORF00914 | 1.552 | conserved hypothetical protein |
| CPK_ORF00133 | 1.504 | conserved hypothetical protein |
| CPK_ORF01102 | 1.457 | conserved domain protein |
| CPK_ORF01015 | 1.292 | conserved hypothetical protein |
| CPK_ORF00938 | 1.281 | conserved hypothetical protein |
| CPK_ORF00547 | 1.279 | IncA family protein (authentic frameshift) |
| CPK_ORF00077 | 1.216 | hypothetical protein |
| CPK_ORF00579 | 1.187 | conserved hypothetical protein |
| CPK_ORF00720 | 1.187 | IncA family protein |
| CPK_ORF00132 | 1.123 | conserved hypothetical protein |
| CPK_ORF00988 | 1.119 | conserved hypothetical protein |
| CPK_ORF00799 | 1.118 | conserved hypothetical protein |
| CPK_ORF00216 | 1.111 | conserved hypothetical protein |
| CPK_ORF00265 | 1.064 | FliH protein |
| CPK_ORF00684 | 1.054 | conserved hypothetical protein |
| CPK_ORF00291 | 1.003 | conserved hypothetical protein |
| CPK_ORF00217 | 0.960 | conserved hypothetical protein |
| CPK_ORF00664 | 0.948 | conserved hypothetical protein |
| CPK_ORF00880 | 0.935 | hypothetical protein |
| CPK_ORF00537 | 0.916 | hypothetical protein |
| CPK_ORF00817 | 0.873 | hypothetical protein |
| CPK_ORF00572 | 0.848 | putative membrane protein |
| CPK_ORF00454 | 0.837 | hypothetical protein |
| CPK_ORF00414 | 0.811 | transcription antitermination factor NusB |
| CPK_ORF00254 | 0.809 | putative trigger factor protein |
| CPK_ORF00285 | 0.792 | SET domain protein |
| CPK_ORF00527 | 0.722 | hypothetical protein |
| CPK_ORF00631 | 0.697 | ribonuclease HII |
| CPK_ORF00337 | 0.685 | conserved hypothetical protein |
| CPK_ORF01038 | 0.681 | conserved hypothetical protein |
| CPK_ORF00018 | 0.638 | nucleoside diphosphate kinase |
| CPK_ORF00576 | 0.626 | conserved hypothetical protein |
| CPK_ORF01118 | 0.621 | conserved hypothetical protein |
| CPK_ORF00131 | 0.589 | conserved hypothetical protein |
| CPK_ORF00841 | 0.587 | conserved hypothetical protein |
| CPK_ORF00692 | 0.586 | IncA family protein |
| CPK_ORF00627 | 0.540 | signal recognition particle protein |
| CPK_ORF00703 | 0.534 | oligopeptide ABC transporter/periplasmic oligopeptide-binding protein OppA |
| CPK_ORF00833 | 0.533 | type III secretion chaperone |
| CPK_ORF00643 | 0.533 | hypothetical protein |
| CPK_ORF00084 | 0.532 | peptide ABC transporter/ATP-binding protein |
| CPK_ORF00078 | 0.500 | conserved hypothetical protein |
| CPK_ORF00873 | 0.500 | hypothetical protein |
| CPK_ORF01030 | 0.481 | ubiquinone/menaquinone biosynthesis methyltransferase |
| CPK_ORF00230 | 0.452 | conserved hypothetical protein |
| CPK_ORF01019 | 0.450 | chaperone protein DnaK |
| CPK_ORF00906 | 0.440 | aminotransferase/class V |
| CPK_ORF00528 | 0.437 | ATP-dependent protease La |
| CPK_ORF00320 | 0.436 | conserved hypothetical protein |
| CPK_ORF00513 | 0.415 | IncA family protein |
| CPK_ORF00867 | 0.414 | GTP-binding protein LepA |
| CPK_ORF00258 | 0.411 | conserved hypothetical protein |
| CPK_ORF00706 | 0.404 | oligopeptide ABC transporter/permease protein OppC |
| CPK_ORF01028 | 0.403 | conserved hypothetical protein TIGR00423 |
| CPK_ORF00448 | 0.402 | conserved hypothetical protein |
| CPK_ORF00302 | 0.397 | transcription-repair coupling factor |
| CPK_ORF01105 | 0.380 | conserved hypothetical protein |
| CPK_ORF00330 | 0.376 | inositol monophosphatase family protein |
| CPK_ORF00732 | 0.374 | conserved hypothetical protein |
| CPK_ORF00282 | 0.374 | putative lipoprotein |
| CPK_ORF01042 | 0.349 | 2-oxo acid dehydrogenase acyltransferase family protein |
| CPK_ORF00777 | 0.344 | renal dipeptidase family protein |
| CPK_ORF00090 | 0.321 | polysaccharide deacetylase domain protein |
| CPK_ORF01031 | 0.318 | hypothetical protein |
| CPK_ORF00004 | 0.316 | putative methyltransferase |
| CPK_ORF01041 | 0.313 | sugar isomerase, KpsF/GutQ family |
| CPK_ORF00052 | 0.307 | UDP-3-O-acyl N-acetylglucosamine deacetylase |
| CPK_ORF00920 | 0.301 | conserved hypothetical protein |
| CPK_ORF00338 | 0.293 | conserved hypothetical protein |
| CPK_ORF00512 | 0.281 | hypothetical protein |
| CPK_ORF00853 | 0.277 | manganese/zinc/iron chelate ABC transporter |
| CPK_ORF01085 | 0.267 | cytidylate kinase |
| CPK_ORF00463 | 0.255 | 3-dehydroquinate synthase |
| CPK_ORF01104 | 0.242 | conserved hypothetical protein |
| CPK_ORF00474 | 0.241 | dihydrodipicolinate reductase |
| CPK_ORF00466 | 0.240 | 3-phosphoshikimate 1-carboxyvinyltransferase |
| CPK_ORF00991 | 0.231 | conserved hypothetical protein |
| CPK_ORF00294 | 0.230 | RNA methyltransferase family protein |
| CPK_ORF01107 | 0.226 | conserved hypothetical protein |
| CPK_ORF00737 | 0.223 | disulfide bond formation protein DsbB |
| CPK_ORF01084 | 0.215 | phosphatidate cytidylyltransferase |
| CPK_ORF00680 | 0.215 | hypothetical protein |
| CPK_ORF00791 | 0.214 | hypothetical protein |
| CPK_ORF00505 | 0.210 | glutamyl-tRNA(Gln) amidotransferase, A subunit |
| CPK_ORF00768 | 0.203 | conserved hypothetical protein |
| CPK_ORF00430 | 0.200 | conserved hypothetical protein |
| CPK_ORF01000 | 0.191 | conserved hypothetical protein |
| CPK_ORF00317 | 0.177 | UDP-N-acetylmuramate--alanine ligase/D-alanine--D-alanine ligase |
| CPK_ORF00889 | 0.175 | putative oxygen-independent coproporphyrinogen III oxidase |
| CPK_ORF00987 | 0.172 | hypothetical protein |
| CPK_ORF00659 | 0.172 | conserved domain protein |
| CPK_ORF00832 | 0.161 | type III secretion regulator YopN/LcrE/InvE/MxiC |
| CPK_ORF00388 | 0.150 | succinyl-CoA synthetase, alpha subunit |
| CPK_ORF00673 | 0.143 | KDO-transferase family protein |
| CPK_ORF00800 | 0.143 | conserved hypothetical protein |
| CPK_ORF00855 | 0.140 | manganese/zinc/iron chelate ABC transporter (MZT) family, ATP-binding protein |
| CPK_ORF00883 | 0.135 | 4-hydroxy-3-methylbut-2-en-1-yl diphosphate synthase |
| CPK_ORF01062 | 0.130 | 2C-methyl-D-erythritol 2, 4-cyclodiphosphate synthase |
| CPK_ORF00076 | 0.125 | conserved hypothetical protein |
| CPK_ORF00989 | 0.111 | putative membrane protein |
| CPK_ORF00409 | 0.105 | ribonucleoside-diphosphate reductase alpha subunit |
| CPK_ORF00924 | 0.101 | conserved hypothetical protein |
| CPK_ORF00205 | 0.099 | hypothetical protein |
| CPK_ORF00762 | 0.098 | RNA modification enzyme, MiaB family |
| CPK_ORF01024 | 0.096 | conserved hypothetical protein |
| CPK_ORF00273 | 0.094 | cadmium-translocating P-type ATPase |
| CPK_ORF00709 | 0.090 | conserved hypothetical protein |
| CPK_ORF00530 | 0.084 | hypothetical protein |
| CPK_ORF00775 | 0.076 | conserved hypothetical protein |
| CPK_ORF00781 | 0.072 | thymidylate kinase |
| CPK_ORF00300 | 0.069 | oxygen-independent coproporphyrinogen III oxidase |
| CPK_ORF00933 | 0.067 | conserved hypothetical protein |
| CPK_ORF00552 | 0.065 | hypothetical protein |
| CPK_ORF00759 | 0.064 | ABC transporter, ATP-binding protein LolD-like protein |
| CPK_ORF00715 | 0.061 | hypothetical protein |
| CPK_ORF01060 | 0.029 | ribosomal protein L21 |
| CPK_ORF01018 | 0.025 | co-chaperone GrpE |
| CPK_ORF00721 | 0.022 | hypothetical protein |
| CPK_ORF00843 | 0.020 | conserved hypothetical protein |
| CPK_ORF00286 | 0.015 | metallo-beta-lactamase family protein |
| CPK_ORF00748 | 0.010 | 6-phosphogluconolactonase |
| CPK_ORF00790 | 0.003 | fructose-bisphosphate aldolase class I |
| CPK_ORF00155 | 0.000 | bacterial transferase hexapeptide repeat protein |
