# Supplementary material for: The Chlamydia psittaci Genome: A Comparative Analysis of Intracellular Pathogens
Source: PLoS One. 2012 Apr 10;7(4):e35097. doi: 10.1371/journal.pone.0035097 (PMC3323650; doi:10.1371/journal.pone.0035097)
Supplement: Table S6 — Predicted type III secreted effectors in Chlamydia caviae GPIC. (DOC) [file pone.0035097.s009.doc]

**Table S6. Predicted type III secreted effectors in *Chlamydia caviae* GPIC**

| ORF | SVM_value | Annotated |
| --- | --- | --- |
| CCA_00170 | 2.342 | conserved hypothetical protein |
| CCA_00425 | 1.516 | conserved hypothetical protein |
| CCA_00550 | 1.469 | inclusion membrane protein A |
| CCA_00389 | 1.418 | conserved hypothetical protein |
| CCA_00062 | 1.350 | conserved hypothetical protein |
| CCA_00941 | 1.310 | conserved hypothetical protein |
| CCA_00367 | 1.258 | conserved hypothetical protein |
| CCA_00490 | 1.223 | inclusion membrane protein C |
| CCA_00261 | 1.221 | conserved hypothetical protein |
| CCA_00908 | 1.192 | conserved hypothetical protein |
| CCA_00955 | 1.190 | conserved hypothetical protein |
| CCA_00536 | 1.069 | hypothetical protein |
| CCA_00424 | 0.987 | hypothetical protein |
| CCA_00221 | 0.963 | conserved hypothetical protein |
| CCA_00549 | 0.952 | methyltransferase, putative |
| CCA_00195 | 0.942 | conserved hypothetical protein |
| CCA_00803 | 0.821 | conserved hypothetical protein |
| CCA_00277 | 0.814 | polymorphic outer membrane protein H family protein |
| CCA_00500 | 0.800 | conserved hypothetical protein |
| CCA_00451 | 0.795 | conserved hypothetical protein |
| CCA_00739 | 0.791 | conserved hypothetical protein |
| CCA_00919 | 0.643 | trigger factor |
| CCA_00645 | 0.638 | hypothetical protein |
| CCA_00622 | 0.630 | hypothetical protein |
| CCA_00658 | 0.619 | signal recognition particle |
| CCA_00474 | 0.610 | hypothetical protein |
| CCA_00296 | 0.603 | conserved hypothetical protein |
| CCA_00722 | 0.597 | conserved domain protein |
| CCA_00772 | 0.596 | N utilization substance protein B, putative |
| CCA_00176 | 0.584 | undecaprenyl diphosphate synthase |
| CCA_00270 | 0.579 | hypothetical protein |
| CCA_00779 | 0.562 | RmuC domain protein |
| CCA_00537 | 0.559 | conserved hypothetical protein |
| CCA_00715 | 0.549 | dihydrodipicolinate reductase |
| CCA_00805 | 0.547 | conserved hypothetical protein |
| CCA_00982 | 0.539 | HAM1 family protein |
| CCA_00063 | 0.511 | conserved hypothetical protein |
| CCA_00291 | 0.509 | conserved hypothetical protein |
| CCA_00766 | 0.496 | YjgP/YjgQ family protein |
| CCA_00558 | 0.480 | putative cytotoxin |
| CCA_00383 | 0.477 | conserved hypothetical protein |
| CCA_00795 | 0.474 | hypothetical protein |
| CCA_00636 | 0.436 | conserved hypothetical protein |
| CCA_00512 | 0.435 | dipeptidase, putative |
| CCA_00723 | 0.424 | 3-phosphoshikimate 1-carboxyvinyltransferase |
| CCA_00245 | 0.421 | conserved hypothetical protein |
| CCA_00250 | 0.420 | hypothetical protein |
| CCA_00180 | 0.419 | conserved hypothetical protein |
| CCA_00139 | 0.396 | conserved hypothetical protein |
| CCA_00470 | 0.395 | conserved hypothetical protein |
| CCA_00911 | 0.382 | UTP--glucose-1-phosphate uridylyltransferase family protein |
| CCA_00797 | 0.379 | hypothetical protein |
| CCA_00353 | 0.373 | conserved hypothetical protein |
| CCA_00916 | 0.370 | phosphoenolpyruvate carboxykinase |
| CCA_00325 | 0.368 | conserved hypothetical protein |
| CCA_00397 | 0.368 | hypothetical protein |
| CCA_00681 | 0.368 | V-type sodium ATP synthase, subunit I |
| CCA_00742 | 0.366 | conserved hypothetical protein |
| CCA_00242 | 0.360 | heat shock protein GrpE |
| CCA_00755 | 0.359 | conserved hypothetical protein |
| CCA_00994 | 0.332 | 5-formyltetrahydrofolate cyclo-ligase, putative |
| CCA_00241 | 0.313 | dnaK protein |
| CCA_00523 | 0.299 | conserved hypothetical protein |
| CCA_01004 | 0.289 | conserved hypothetical protein |
| CCA_00015 | 0.279 | conserved hypothetical protein |
| CCA_00741 | 0.277 | conserved hypothetical protein |
| CCA_00194 | 0.275 | oxidoreductase |
| CCA_00050 | 0.268 | conserved hypothetical protein |
| CCA_00219 | 0.260 | carbohydrate isomerase, KpsF/GutQ family |
| CCA_00603 | 0.256 | peptide ABC transporter, permease protein |
| CCA_00297 | 0.252 | conserved hypothetical protein |
| CCA_00634 | 0.246 | hypothetical protein |
| CCA_00135 | 0.233 | conserved hypothetical protein TIGR00095 |
| CCA_00153 | 0.231 | conserved hypothetical protein |
| CCA_00702 | 0.225 | hypothetical protein |
| CCA_00016 | 0.219 | conserved hypothetical protein |
| CCA_00430 | 0.218 | hypothetical protein |
| CCA_00899 | 0.217 | cadmium-translocating P-type ATPase |
| CCA_00350 | 0.214 | conserved hypothetical protein |
| CCA_00070 | 0.209 | conserved hypothetical protein |
| CCA_00457 | 0.207 | type III secretion chaperone |
| CCA_00331 | 0.205 | DNA polymerase III, gamma subunit |
| CCA_00452 | 0.200 | conserved hypothetical protein |
| CCA_00400 | 0.198 | nifS protein, putative |
| CCA_00783 | 0.194 | succinyl-Coa synthetase, alpha chain |
| CCA_00423 | 0.178 | gcpE protein |
| CCA_00475 | 0.175 | glycogen phosphorylase |
| CCA_00279 | 0.175 | polymorphic outer membrane protein G family protein |
| CCA_00820 | 0.173 | ribosomal 5S rRNA E-loop binding protein Ctc/L25/TL5 |
| CCA_00157 | 0.171 | sensor histidine kinase |
| CCA_00433 | 0.163 | GTP-binding protein LepA |
| CCA_00667 | 0.154 | conserved hypothetical protein |
| CCA_00268 | 0.136 | 1,4-alpha-glucan branching enzyme |
| CCA_00545 | 0.136 | amino acid ABC transporter, ATP-binding protein |
| CCA_00413 | 0.132 | conserved hypothetical protein |
| CCA_00124 | 0.122 | replicative DNA helicase |
| CCA_01001 | 0.120 | RNA polymerase sigma factor, sigma-70 family |
| CCA_00860 | 0.117 | conserved hypothetical protein |
| CCA_00289 | 0.117 | glutamyl-tRNA(Gln) amidotransferase, B subunit |
| CCA_00701 | 0.115 | conserved hypothetical protein |
| CCA_00905 | 0.110 | nifS protein, putative |
| CCA_00525 | 0.108 | conserved hypothetical protein |
| CCA_00334 | 0.098 | hypothetical protein |
| CCA_00557 | 0.098 | hypothetical protein |
| CCA_00776 | 0.088 | ribonucleoside-diphosphate reductase, beta subunit |
| CCA_00421 | 0.084 | 2-oxoglutarate dehydrogenase, E2 component, dihydrolipoamide succinyltransferase |
| CCA_00862 | 0.084 | KH domain protein |
| CCA_00599 | 0.081 | peptide ABC transporter, periplasmic binding protein |
| CCA_00284 | 0.074 | polymorphic outer membrane protein G family protein/autotransporter |
| CCA_00438 | 0.071 | ABC transporter, ATP-binding protein |
| CCA_00263 | 0.068 | conserved hypothetical protein |
| CCA_00304 | 0.060 | deoxyxylulose-5-phosphate synthase |
| CCA_00674 | 0.059 | hypothetical protein |
| CCA_00668 | 0.049 | conserved hypothetical protein |
| CCA_00502 | 0.047 | ABC transporter, permease protein |
| CCA_00174 | 0.045 | cytidylate kinase |
| CCA_00256 | 0.041 | conserved hypothetical protein |
| CCA_00619 | 0.040 | conserved domain protein |
| CCA_00608 | 0.037 | pyrophosphate--fructose 6-phosphate 1-phosphotransferase |
| CCA_00995 | 0.036 | recA protein |
| CCA_00329 | 0.034 | phosphoenolpyruvate-protein phosphotransferase |
| CCA_00844 | 0.025 | conserved hypothetical protein |
| CCA_00727 | 0.017 | 3-dehydroquinate dehydratase/shikimate 5-dehydrogenase |
| CCA_00598 | 0.016 | conserved hypothetical protein TIGR00250 |
| CCA_00915 | 0.014 | conserved hypothetical protein |
| CCA_00152 | 0.013 | conserved hypothetical protein |
| CCA_00508 | 0.012 | thymidylate kinase |
| CCA_00938 | 0.010 | type III secretion inner membrane protein SctR |
| CCA_00371 | 0.006 | conserved hypothetical protein |
| CCA_00342 | 0.002 | ribonuclease III |
| CCA_00756 | 0.002 | conserved hypothetical protein |
| CCA_00211 | 0.000 | transcriptional regulator, putative |
