# Supplementary material for: The Chlamydia psittaci Genome: A Comparative Analysis of Intracellular Pathogens
Source: PLoS One. 2012 Apr 10;7(4):e35097. doi: 10.1371/journal.pone.0035097 (PMC3323650; doi:10.1371/journal.pone.0035097)
Supplement: Table S7 — Type III secreted effectors in Chlamydia psittaci 6BC predicted by EffectiveT3 ( http://www.effectors.org/ ) (DOC) [file pone.0035097.s010.doc]

**Table S7. Type III secreted effectors in *Chlamydia psittaci* 6BC predicted by EffectiveT3 (**<http://www.effectors.org/>**)**

| gene | protein | is T3 secreted | T3 Score | SVM decision value |
| --- | --- | --- | --- | --- |
| CPSIT_0192 | TARP | + | 1 | 1.654666 |
| CPSIT_0429 | hypothetical protein | + | 1 | 1.308868 |
| CPSIT_0580 | Putative inner membrane protein | + | 1 | 1.224083 |
| CPSIT_0074 | hypothetical serine-rich protein | + | 1 | 1.220956 |
| CPSIT_0532 | inclusion membrane protein B | + | 1 | 1.086837 |
| CPSIT_0606 | adherence factor | + | 1 | 1.061218 |
| CPSIT_0594 | inclusion membrane protein A | + | 1 | 1.004527 |
| CPSIT_0431 | Putative membrane protein | + | 1 | 0.951884 |
| CPSIT_0846 | putative TMH-family membrane protein | + | 1 | 0.895848 |
| CPSIT_0844 | putative TMH-family membrane protein | + | 1 | 0.864210 |
| CPSIT_0749 | hypothetical protein | + | 1 | 0.768785 |
| CPSIT_0785 | hypothetical serine-rich protein | + | 1 | 0.737329 |
| CPSIT_0853 | Putative membrane protein | + | 0.99993 | 0.706989 |
| CPSIT_0656 | putative integral membrane protein | + | 1 | 0.551655 |
| CPSIT_0767 | 3-phosphoshikimate 1-carboxyvinyltransferase aroA | + | 1 | 0.506393 |
| CPSIT_0271 | hypothetical protein | + | 1 | 0.498651 |
| CPSIT_0350 | hypothetical protein | + | 1 | 0.487864 |
| CPSIT_0316 | polymorphic outer membrane protein G family protein pmp18G | + | 1 | 0.486637 |
| CPSIT_0139 | replicative DNA helicase dnaB | + | 1 | 0.445357 |
| CPSIT_0247 | hypothetical protein | + | 1 | 0.341109 |
| CPSIT_0855 | hypothetical protein | + | 1 | 0.334555 |
| CPSIT_0335 | 1-deoxy-D-xylulose-5-phosphate synthase dxs | + | 1 | 0.319342 |
| CPSIT_0965 | UTP--glucose-1-phosphate uridylyltransferase | + | 1 | 0.307761 |
| CPSIT_1011 | hypothetical protein | + | 1 | 0.278525 |
| CPSIT_0179 | hypothetical protein | + | 1 | 0.273594 |
| CPSIT_0541 | hypothetical protein | + | 1 | 0.265470 |
| CPSIT_0788 | hypothetical protein | + | 1 | 0.225887 |
| CPSIT_0959 | cystein desulfurase | + | 1 | 0.181195 |
| CPSIT_0368 | hypothetical protein | + | 1 | 0.175724 |
| CPSIT_1012 | outer protein D1, copD1 | + | 1 | 0.121398 |
| CPSIT_0382 | hypothetical protein | + | 0.99995 | 0.118126 |
| CPSIT_0366 | hypothetical protein | + | 1 | 0.094030 |
| CPSIT_0700 | signal recognition particle protein ffh | + | 1 | 0.091777 |
| CPSIT_0174 | hypothetical protein | + | 1 | 0.075889 |
| CPSIT_0787 | hypothetical protein | + | 1 | 0.015362 |
| CPSIT_0545 | hypothetical protein | + | 0.99999 | - |
| CPSIT_0604 | hypothetical protein | + | 0.99999 | - |
| CPSIT_0557 | hypothetical protein | + | 0.99999 | - |
| CPSIT_0432 | hypothetical protein | + | 0.99998 | - |
| CPSIT_0363 | DNA polymerase III subunits gamma and tau, dnaX | + | 0.99997 | - |
| CPSIT_0648 | putative ester hydrolase | + | 0.99997 | - |
| CPSIT_0053 | uridylate kinase pyrH | + | 0.99997 | - |
| CPSIT_0401 | S-adenosyl-methyltransferase mraW | + | 0.99996 | - |
| CPSIT_0312 | polymorphic outer membrane protein G family protein pmp15G | + | 0.99995 | - |
| CPSIT_0373 | phosphoglucomutase/phosphomannomutase family protein | + | 0.99993 | - |
| CPSIT_0717 | hypothetical protein | + | 0.99992 | - |
| CPSIT_0212 | hypothetical protein | + | 0.99992 | - |
| CPSIT_0203 | single-stranded-DNA-specific exonuclease recJ | + | 0.99991 | - |
| CPSIT_0252 | diaminopimelate epimerase dapF | + | 0.99991 | - |
| CPSIT_0361 | phosphoenolpyruvate-protein phosphotransferase | + | 0.9999 | - |
| CPSIT_0287 | hypothetical protein | + | 1 | - |
| CPSIT_0085 | DNA polymerase III subunit alpha dnaE | + | 1 | - |
| CPSIT_0608 | MAC/Perforin domain-containing protein | + | 1 | - |
| CPSIT_0321 | putative inner membrane protein | + | 1 | - |
| CPSIT_0468 | RNA polymerase sigma factor | + | 1 | - |
| CPSIT_0986 | branched-chain amino acid transport system II carrier protein brnQ | + | 1 | - |
| CPSIT_0277 | hypothetical protein | + | 1 | - |
| CPSIT_0365 | putative inner membrane protein | + | 1 | - |
| CPSIT_0766 | hypothetical protein | + | 1 | - |
| CPSIT_0183 | hypothetical protein | + | 1 | - |
| CPSIT_0531 | inclusion membrane protein C, incC | + | 1 | - |
| CPSIT_0338 | exodeoxyribonuclease VII large subunit, xseA | + | 1 | - |
| CPSIT_0417 | hypothetical protein | + | 1 | - |
| CPSIT_0804 | hypothetical protein | + | 1 | - |
| CPSIT_1043 | hypothetical protein | + | 1 | - |
| CPSIT_0803 | hypothetical protein | + | 1 | - |
| CPSIT_0802 | hypothetical protein | + | 1 | - |
| CPSIT_0311 | polymorphic outer membrane protein G family protein, pmp14G | + | 1 | - |
